# Phylogeny and multiple independent whole‐genome duplication events in the Brassicales

**DOI:** 10.1002/ajb2.1514

**Published:** 2020-08-24

**Authors:** Makenzie E. Mabry, Julia M. Brose, Paul D. Blischak, Brittany Sutherland, Wade T. Dismukes, Christopher A. Bottoms, Patrick P. Edger, Jacob D. Washburn, Hong An, Jocelyn C. Hall, Michael R. McKain, Ihsan Al‐Shehbaz, Michael S. Barker, M. Eric Schranz, Gavin C. Conant, J. Chris Pires

**Affiliations:** ^1^ Division of Biological Sciences and Christopher S. Bond Life Sciences Center University of Missouri Columbia Missouri 65211 USA; ^2^ Department of Ecology and Evolutionary Biology University of Arizona Tucson Arizona 85719 USA; ^3^ Informatics Research Core Facility and Christopher S. Bond Life Sciences Center University of Missouri Columbia Missouri 65211 USA; ^4^ Department of Horticulture Michigan State University East Lansing Michigan 48824 USA; ^5^ Plant Genetics Research Unit USDA‐ARS Columbia Missouri 65211 USA; ^6^ Department of Biological Sciences University of Alberta Edmonton T6G 2E9 Canada; ^7^ Department of Biological Sciences The University of Alabama Tuscaloosa Alabama 35401 USA; ^8^ Missouri Botanical Garden St. Louis Missouri 63110 USA; ^9^ Wageningen University and Research Wageningen The Netherlands; ^10^ Bioinformatics Research Center Program in Genetics and Department of Biological Sciences North Carolina State University Raleigh North Carolina 27695 USA

**Keywords:** Brassicales, Capparaceae, Cleomaceae, phylogeny, phylo‐transcriptomics, Resedaceae, whole‐genome duplication

## Abstract

**Premise:**

Whole‐genome duplications (WGDs) are prevalent throughout the evolutionary history of plants. For example, dozens of WGDs have been phylogenetically localized across the order Brassicales, specifically, within the family Brassicaceae. A WGD event has also been identified in the Cleomaceae, the sister family to Brassicaceae, yet its placement, as well as that of WGDs in other families in the order, remains unclear.

**Methods:**

Phylo‐transcriptomic data were generated and used to infer a nuclear phylogeny for 74 Brassicales taxa. Genome survey sequencing was also performed on 66 of those taxa to infer a chloroplast phylogeny. These phylogenies were used to assess and confirm relationships among the major families of the Brassicales and within Brassicaceae. Multiple WGD inference methods were then used to assess the placement of WGDs on the nuclear phylogeny.

**Results:**

Well‐supported chloroplast and nuclear phylogenies for the Brassicales and the putative placement of the Cleomaceae‐specific WGD event Th‐ɑ are presented. This work also provides evidence for previously hypothesized WGDs, including a well‐supported event shared by at least two members of the Resedaceae family, and a possible event within the Capparaceae.

**Conclusions:**

Phylogenetics and the placement of WGDs within highly polyploid lineages continues to be a major challenge. This study adds to the conversation on WGD inference difficulties by demonstrating that sampling is especially important for WGD identification and phylogenetic placement. Given its economic importance and genomic resources, the Brassicales continues to be an ideal group for assessing WGD inference methods.

The Brassicales is an economically important order of flowering plants, home to crop species (kale, broccoli, cabbage, cauliflower, papaya, capers, and canola) as well as several model species, including *Arabidopsis* spp. There are currently 17 accepted families within the order (APG IV, 2016), with the Brassicaceae being the most well‐studied family due to its many crop and model plant species. Together, the Brassicaceae and the closely related Cleomaceae and Capparaceae contain 94% of the species diversity in the order (Edger et al., 2015). Sister to these three families is an unresolved clade of four families: Tovariaceae, Gyrostemonaceae, Resedaceae, and Pentadiplandraceae. This clade is followed by Emblingiaceae, [Salvadoraceae + Bataceae], Koeberliniaceae], Limnanthaceae, Setchellanthaceae, [Caricaceae + Morginaceae], and [Tropaeolaceae + Akaniaceaeae] (Appendix S1; see the Supplemental Data with this article; APG IV, 2016). The order diverged from other rosids ~103 mya and represents 2.2% of the total extant core eudicot diversity (Magallon et al., 1999; Cardinal‐McTeague et al., 2016). Previous research has identified multiple whole‐genome duplication (WGD) events across the order using a variety of comparative methods, including genomics, transcriptomics, and molecular cytogenetics (Vision et al., 2000; Schranz and Mitchell‐Olds, 2006; Barker et al., 2009; Cheng et al., 2013; Kagale et al., 2014; Edger et al., 2015, 2018; Mandáková et al., 2017; Lysak, 2018; One Thousand Plant Transcriptomes Initiative, 2019; Appendix S1). Four of the most studied events include one near the base of the order (At‐β; Edger et al., 2015, 2018), an event at the base of the Brassicaceae (At‐ɑ; Vision et al., 2000; Haudry et al., 2013; Edger et al., 2015), a triplication at the base of the tribe Brassiceae in the Brassicaceae (Lysak et al., 2005; Tang et al., 2012), and an unplaced event within the Cleomaceae (Th‐ɑ; Schranz and Mitchell‐Olds, 2006; Barker et al., 2009).

Within the Brassicales, the Brassicaceae has the largest number of accepted species (>4000; BrassiBase). It contains the model plant *Arabidopsis thaliana* (Arabidopsis Genome Initiative, 2000) as well as important crops of the *Brassica* and *Raphanus* groups. Its clades have been placed into three major lineages (Lineage I, Lineage II, and Lineage III; Beilstein et al., 2006), with notable named clades acknowledged more recently (i.e., Clade C; Huang et al., 2016; Nikolov et al., 2019). The relationships among these lineages and clades are unclear. Besides elucidating the relationships within the Brassicaceae, another major area of research has focused on the considerable glucosinolate diversity within the family (Kliebenstein et al., 2001; Ratzka et al., 2002; Züst et al., 2018; Blažević et al., 2019), including the impact of WGD events on the glucosinolate chemical structures (Edger et al., 2015; Barco and Clay, 2019).

Sister to the Brassicaceae is the Cleomaceae. A mostly herbaceous family of ~270 species of pantropical plants, it diverged from the Brassicaceae ~40 mya (Edger et al., 2015). The Cleomaceae displays a much wider range of floral morphologies than its sister family, a characteristic that has been the focus of several recent studies (Bhide et al., 2014; Brock, 2014; Bayat et al., 2018). This family is unique among the Brassicales for containing species that utilize C_4_ photosynthesis (*Gynandropsis gynandra* and *Coalisina angustifolia*, formerly *Cleome angustifolia*; Feodorova et al., 2010) as well as, though not unique to Cleomaceae (Schlüter et al., 2016), a C_3_‐C_4_ intermediate (*Coalisina paradoxa*, formerly *C. paradoxa*; van den Bergh et al., 2014). Cleomaceae is known to have undergone at least one independent polyploidy event that occurred after the split from the Brassicaceae, named Th‐α (after *Tarenaya hassleriana*), and has been dated to ~13.7 mya (Schranz and Mitchell‐Olds, 2006; Barker et al., 2009; Cheng et al., 2013). The analyses for this identification and placement used only partial genomic fragments, ESTs, or a single genome. It was subsequently determined that the Th‐α event is shared with the species *G. gynandra*, a C_4_ species (van den Bergh et al., 2014), but not with *Cleome violacea*, *Arivela viscosa*, or *Polanisia trachysperma* (Emery et al., 2018; One Thousand Plant Transcriptomes Initiative, 2019). The precise phylogenetic location of Th‐α remains a mystery (van den Bergh et al., 2014; Bayat et al., 2018).

The Capparaceae—a mostly woody tropical family of 450 species—is less studied than either of its two sister families, Brassicaceae and Cleomaceae. Like the Cleomaceae, the Capparaceae is very diverse in its floral morphology (Endress, 1992), and, like other members of the order (with the exception of *Koeberlinia spinosa*; Tobe and Raven, 2008), it produces glucosinolates but shares the production of unique methyl‐glucosinolates with only the Cleomaceae (Hall et al., 2002; Mithen et al., 2010). In this group is the economically important species *Capparis spinosa*, or capers. Recent work using chromosome counts hypothesized that the Capparaceae and a more distant family, the Resedaceae, may possess unique WGD events (Lysak, 2018). Members of the Resedaceae, a relatively small clade of ~85 species, are mostly distributed across Europe, the Middle East, and Africa, with one taxon occurring in North America (*Oligomeris linifolia*) due to a long‐distance dispersal event (Martín‐Bravo et al., 2007, 2009; Cardinal‐McTeague et al., 2016).

To infer phylogenetic relationships within the Brassicales, we used phylo‐transcriptomics, a quickly evolving subdiscipline of phylogenomics that uses RNA‐seq data as the basis of its inferences (Dunn et al., 2008; McKain et al., 2012; Yang et al., 2015; Washburn et al., 2017; Unruh et al., 2018; Godden et al., 2019). Transcriptomics gives access to many more nuclear genes than traditional PCR‐based approaches and is less expensive than sequencing an entire genome. RNA‐seq data also allows for assessing gene and genome duplication events (Baker et al., 2009; McKain et al., 2012). One difficulty with using transcriptomes for phylogenetic inference is an inability to determine orthology (Dunn et al., 2013; Yang and Smith 2014; Washburn et al., 2017; Emms and Kelly, 2019). Several methods have been developed to address this problem, including those that aim to identify orthogroups, or sets of genes descended from a single gene in the last common ancestor of the group or species of interest (Duarte et al., 2010; Emms and Kelly, 2015). OrthoFinder version 2 (Emms and Kelly, 2019) offers improvements in both orthogroup inference accuracy and in computational speed, especially when using Diamond (an alternative to BLAST; Buchfink et al., 2015). Together, these methods have enabled phylo‐transcriptomics to be extremely useful for inferring species relationships, understanding gene evolution, and elucidating WGD events.

The Brassicales are an intriguing group for the study of polyploidy. With well‐established WGD events across the Brassicaceae, including At‐ɑ at the base (Vision et al., 2000; Edger et al., 2015) and the identification of a unique and more recent, albeit unplaced, event in the Cleomaceae (Th‐ɑ; Schranz and Mitchell‐Olds, 2006; Barker et al., 2009; Cheng et al., 2013), one wonders what makes this group unique. Here, we aim to answer the remaining questions on the placement of WGD events, including Th‐ɑ, using phylo‐transcriptomics, with a focus on sampling the Brassicaceae and Cleomaceae and additional sampling of the Capparaceae, Resedaceae, Bataceae, Caricaceae, and Moringaceae families. We ask if Th‐ɑ is shared across the Cleomaceae or if the family, like the Brassicaceae, is characterized by multiple events. We also test the recent hypothesis that the Resedaceae and the Capparaceae possess independent WGD events (Lysak, 2018). We demonstrate that the Brassicales are a powerful resource for the study of WGD and an important group to test how WGD correlates with variation in floral morphology, photosynthesis types, metabolism (especially glucosinolates), and other traits of interest.

## MATERIALS AND METHODS

### Taxon sampling

Sampling of 74 taxa from 57 genera across the Brassicales spanned seven families (Brassicaceae, Cleomaceae, Capparaceae, Resedaceae, Bataceae, Moringaceae, Caricaceae), with a focus on the Brassicaceae (48 taxa) and Cleomaceae (17 taxa) (Appendix S2). Seeds were grown at the University of Missouri or the University of Alberta in a sterile growth chamber environment. At maturity, but before flowering, leaf tissue was collected for both RNA and DNA extraction.

### DNA and RNA isolation and sequencing

DNA was extracted from leaf tissue for 69 of the 74 taxa using a DNeasy Plant Kit (Qiagen, Germantown, Maryland, USA). To increase yield, slight modifications to the manufacturer’s protocol included increasing lysis buffer incubation time to 1 h and using 25 µL of buffer to elute the final sample. TruSeq library preparation (Illumina, San Diego, California, USA) and genome survey sequencing (GSS, also known as skim sequencing) on a NextSeq instrument were carried out at the University of Missouri, resulting in 2 × 150 bp reads.

RNA from leaf tissue was collected and immediately flash frozen using liquid nitrogen. For 38 samples, RNA was isolated using an Invitrogen PureLink RNA Mini Kit (Thermo Fisher Scientific, Carlsbad, California) followed by TruSeq library preparation and sequencing on the NextSeq, resulting in 2 × 75 bp reads (Appendix S3). For 16 samples, RNA was isolated using an Invitrogen PureLink RNA Mini Kit then sequenced on an Illumina HiSeq instrument, resulting in 2 × 100 bp reads (Appendix S3). For 17 samples, RNA was sequenced on a HiSeq for 2 × 100 bp reads but using a Qiagen RNeasy Plant Kit for RNA isolation (Appendix S3). Two samples were isolated using a ThermoFisher Invitrogen PureLink RNA Mini Kit) and sequenced on a HiSeq for 2 × 250 bp reads (Appendix S3). All sequencing and library preparation for the above samples was performed by the University of Missouri DNA Core Facility.

At the University of Alberta, the sample *Cleomella serrulata* had tissue pooled from leaves, apical meristematic tissue, and floral tissue of different developmental stages including small, medium, and large buds and open flowers from two plants. Total RNA was extracted using a Qiagen RNeasy Plant Mini Kit following the manufacturer’s protocol, then treated with DNAse I (New England Biolabs, Ipswich, Massachusetts, USA) for 30 min at 37°C to remove residual DNA from the total RNA. Sequencing was conducted by Plateforme d’analyses génomique (l’Université Laval, Quebec City, Quebec, Canada) with Illumina TruSeq RNASeq for library preparation and Illumina for sequencing for paired‐end 2 × 100 bp reads.

### Chloroplast assembly, alignment, and phylogenomics

To verify identification of taxa, an analysis was performed with two previously published chloroplast genes, *matK* and *ndhF*, for 91 taxa (Hall, 2008) plus 66 samples from this study. The two chloroplast genes were annotated and extracted from de novo whole‐chloroplast sequences (discussed below) using Geneious version 8.1.9 (Kearse et al., 2012). We were unable to annotate and extract *ndhF* for the taxon *Batis maritima*. Alignment of resulting genes was performed in MAFFT version 7 (Katoh et al., 2002) and cleaned with Phyutility version 2.7.1 (Smith and Dunn, 2008) using the parameter *‐clean 0.5*. For maximum likelihood (ML) phylogenetic inference, RAxML version 8 (Stamatakis, 2014) was run with a separate partition for each gene, GTRGAMMA as the model, and 1000 bootstrap replicates.

To assemble the de novo chloroplasts sequences from the GSS data, we used Fast‐Plast version 1.2.8 (McKain and Wilson, 2017). This method utilizes Trimmomatic version 0.35 (Bolger et al., 2014) to clean the reads of adaptors using a Phred score of 33 (Bowtie2 version 2.3.4.3; Langmead and Salzberg, 2012) to separate chloroplast reads by mapping them to a reference database of angiosperm chloroplasts, followed by both SPAdes version 3.13.0 (Bankevich et al., 2012) and “afin” to assemble reads (https://github.com/mrmckain/Fast‐Plast/tree/master/afin). For 13 samples that would not assemble with the default options, the *‐‐subsample* option yielded successful assemblies (Appendix S2). Since we obtained only partial regions of the chloroplast genomes for *Polanisia dodecandra*, *Farsetia aegyptia*, and *Cardamine hirsuta*, these samples were excluded from the downstream analyses. Following assembly, MAFFT was used to align the large single copy (LSC), the small single copy (SSC), and one copy of the inverted repeat (IR). Alignments were cleaned with Phyutility using the parameter *‐clean 0.5*. Maximum likelihood phylogenomic inference was performed in RAxML with partitions for each region, GTRGAMMA as the model, and 1000 bootstrap replicates.

### Transcriptome assembly, alignment, and phylogenomics

For transcriptome analyses, reads were trimmed with Trimmomatic using the parameters *SLIDINGWINDOW:4:5*, *LEADING:5*, *TRAILING:5*, and *MINLEN:25*, followed by assembly with Trinity version 2.2 (Grabherr et al., 2011). The resulting de novo transcriptomes were checked for completeness in BUSCO version 3 (Simão et al., 2015; Waterhouse et al., 2017) and compared to the Embryophyta database. Transcriptomes were translated to protein sequences by extracting the longest open reading frame, and coding regions were predicted using TransDecoder version 3.0 (github.com/TransDecoder/TransDecoder). Finally, orthology was inferred in OrthoFinder version 2.2.6, first using the parameter *‐S diamond* (Buchfink et al., 2015) and then the parameter *‐M msa ‐ot* for multiple sequence alignments and only trees. Using custom scripts, alignments were filtered for 80% taxon occupancy (github.com/MU‐IRCF/filter_by_ortho_group) and alignment quality, allowing for only 40% gaps (github.com/MU‐IRCF/filter_by_gap_fraction). To estimate gene trees using ML inference, RAxML was used (Stamatakis, 2014) under the PROTCATWAG model and 100 bootstrap replicates. Gene trees were analyzed in PhyloTreePruner version 1.0 (Kocot et al., 2013) to remove any paralogous genes by using a cutoff of 10 for the minimum number of taxa required to keep a group. Resulting alignments were then used to estimate final gene trees with RAxML under the PROTCATWAG model and 100 bootstrap replicates. Species tree estimation for the Brassicales was performed with ASTRAL‐III version 5.6.1 (Zhang et al., 2018) and included the parameter *‐t 2* to assess discordance among gene trees. Species tree analyses were also performed at the family level (Brassicaceae, Cleomaceae, Capparaceae, [Resedaceae + Bataceae + Moringaceae + Cariacacae]; Appendix S4).

### Whole‐genome duplication

To estimate the phylogenetic placement of whole‐genome duplications, we used PUG version 2.1 (github.com/mrmckain/PUG) to query putative paralogs over multiple gene trees with the estimated ASTRAL‐III tree as the input species tree. For each analysis, we used the original ML gene trees before running them through PhyloTreePruner (i.e., gene trees with all duplicates retained), the ASTRAL‐III tree (rooted and with node labels removed), and parameters *‐‐estimate_paralogs* and *‐‐outgroups Carica_papaya, Moringa_oleifera* as input. Output duplicate gene counts were used for nodes with bootstrap values ≥80%.

As another confirmation of duplication events, we constructed histograms giving the distribution of the synonymous divergence (*K*_s_) between paralogs in each transcriptome. This method allows for the potential identification of peaks in the distribution that may be indicative of a WGD event. The position of the peak along the *K*_s_ axis provides an estimate of when the event occurred. Typically, the peak closest to time zero (or *K*_s_ ~ 0) corresponds to recent tandem duplicates, not relevant to WGD events. Plots of *K*_s_ distributions were made for all taxa in FASTKs version 1.1 (github.com/mrmckain/FASTKs), as described in McKain et al. (2016), and in DupPipe, following Barker et al. (2010). R version 3.5.1 (R Core Team, 2018) was used to estimate normal mixture models for *K*_s_ values using the “mclust” package version 5.0.2 (Fraley and Raftery, 2002; Fraley et al., 2012). To assess for the best number of peaks to explain the data, we tested one to four components for each mixture model. We chose the component with the lowest Bayesian information criterion (BIC) score as the best fit (Appendix S5).

Ortholog divergence was estimated using OrthoPipe, as described in Barker et al. (2010). Using the estimated ortholog divergence and the DupPipe *K*_s_ estimates, we bookended the positions of potential events by comparing when species diverged to the mean paralog divergence of an estimated WGD event: if the ortholog divergence between pairs of species is greater (larger *K*_s_ value) than the paralog divergence of a WGD event, then the species do not share the event; if the ortholog divergence between species is less than the WGD paralog divergence, then the species share the proposed event.

## RESULTS

### Sequence matrices

DNA read pools range in size from 6,637,717 to 13,335,392 reads per sample. After assembly of complete chloroplasts, the inferred genomes for the 66 taxa range in length from 137,110 to 160,272 bp. The LSC, SSC, and IR regions were isolated and aligned separately, with total alignment lengths of 84,350 bp, 17,931 bp, and 26,500 bp, respectively. The analysis of two previously published copies of *matK* and *ndhF*, in combination with our own data, resulted in alignment lengths of 1521 and 985 bp for each gene, respectively. Both chloroplast analyses had 100% occupancy for taxa included.

RNA read pools range in size from 5,555,024 to 59,723,745 reads per sample, with an average of 22,520,865 reads per sample. To check the completeness of transcriptomes, the assemblies were run though BUSCO. All assemblies had >66% complete genes, with <12% of genes missing or fragmented (Appendix S6). OrthoFinder version 2.2.6 recovered 47,600 orthogroups across the Brassicales. Filtering for 80% taxon occupancy (59/74 taxa) yielded 10,968 orthogroups. After filtering for alignment quality by allowing for only 40% gaps, we recovered 2663 orthogroups. Pruning trees for any remaining paralogs, by using a minimum of 10 taxa as a cutoff, resulted in 1284 orthogroups, which were then used for species tree inference. Following the steps above for each family (Brassicaceae, Capparaceae, Cleomaceae, and Resedaceae + Bataceae + Moringaceae + Caricaceae), we recovered 2100, 10,214, 3626, and 8476 orthogroups, respectively (Appendix S4).

### Chloroplast phylogenomics of the Brassicales

The analysis of the chloroplast genes *matK* and *ndhF*, the 91 taxa from the study by Hall (2008), and our 66 samples identified some inconsistencies of species placement but recovered the same overall relationships as published for other chloroplast phylogenies of the Brassicales (Hall, 2008; Cardinal‐McTeague et al., 2016; Edger et al., 2018; Appendix S7). Species sampled in Hall (2008) and in the present study that are not recovered as sister to one another include *Stanleya pinnata*, *Cleomella lutea*, *Andinocleome pilosa*, and *Capparis tomentosa*. The lack of congruence for species placement may be due to species being mislabeled (e.g., *Cleomella lutea*) or species being more genetically diverse than previously thought. Due to this uncertainty in taxon identification, we refer to these samples as Brassicaceae sp., *Polanisia* sp., Cleomaceae sp., and Capparaceae sp., respectively.

For the whole‐chloroplast analyses, using just one copy of the IR, all nodes except four were recovered with 70% bootstrap support or better and with a topology largely congruent with previous studies (Hall, 2008; Cardinal‐McTeague et al., 2016; Edger et al., 2018). This agreement includes a clade of *Moringa oleifera* and *Carica papaya* sister to a clade of [Bataceae + Resedaceae + Capparaceae + Cleomaceae + Brassicaceae], followed by Bataceae sister to [Resedaceae + Capparaceae + Cleomaceae + Brassicaceae], Resedaceae sister to [Capparaceae + Cleomaceae + Brassicaceae], and Capparaceae sister to [Cleomaceae + Brassicaceae] (Appendix S8). The relationships among the major lineages within the Brassicaceae were also in agreement with previous studies (Guo et al., 2017). We recovered *Aethionema arabicum* as sister to the rest of the family, followed by Lineage I sister to [Lineage III + Clade C + Lineage II and Expanded Lineage II] and Lineage III sister to [Clade C + Lineage II and expanded Lineage II]. Relationships within the Cleomaceae were congruent with previous studies (Hall, 2008; Patchell et al., 2014), with *Cleome* sensu stricto (after Patchell et al., 2014) sister to *Polanisia* plus the rest of the family. Most likely due to sampling, our relationships among the Capparaceae were not congruent with previous studies (Hall, 2008; Tamboli et al., 2018). Previous studies with more sampling recovered *Boscia* sp. sister to *Cadaba*, while we recovered *Boscia* as sister to *Capparis*.

### Phylo‐transcriptiomics of the Brassicales

**Figure 1:**
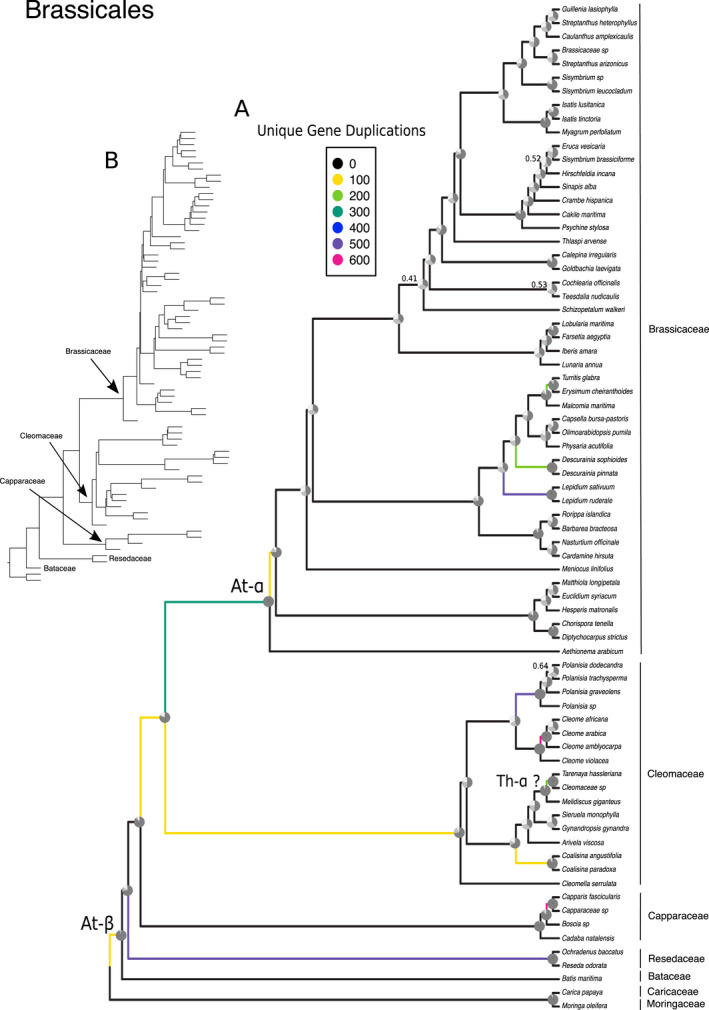
*ASTRAL‐III species phylogeny and whole‐genome duplication events of the Brassicales. (A) Species tree with branch lengths proportional and discordance plotted as pie charts at nodes. Dark gray denotes proportion of gene trees agreeing with main topology; lighter gray denotes proportion of gene trees agreeing with first alternative topology; lightest gray denotes proportion of gene trees agreeing with second alternative topology. Previously inferred events (At‐ɑ and At‐β) and possible placement of Th‐ɑ are indicated. Branch color denotes number of unique gene duplications as determined by PUG (github.com/mrmckain/PUG). Support values are indicated if <0.7 local posterior probability. (B) Coalescent‐based species tree with branch lengths.*

Analysis of nuclear data from the transcriptome with ASTRAL‐III recovers a well‐resolved tree with all nodes but four having a local posterior probability ≥0.7 (Fig. 1). The overall relationships of the families and major lineages were congruent with previous studies using transcriptomics (Edger et al., 2015). As with the whole‐chloroplast phylogeny, we recover a clade of *Moringa oleifera* and *Carica papaya* sister to a clade of [Bataceae + Resedaceae + Capparaceae + Cleomaceae + Brassicaceae], Bataceae sister to [Resedaceae + Capparaceae + Cleomaceae + Brassicaceae], Resedaceae sister to [Capparaceae + Cleomaceae + Brassicaceae], and Capparaceae sister to [Cleomaceae + Brassicaceae]. Within Brassicaceae, the major lineages were recovered, as supported by previous literature (Huang et al., 2016; Nikolov et al., 2019), with *Aethionema arabicum* as sister to the rest of the family, followed by Lineage III sister to [Lineage I + Clade C + Lineage II and expanded Lineage II], and Lineage I sister to [Clade C + Lineage II and expanded Lineage II]. Within Cleomaceae, the relationships were mostly congruent with previous nuclear phylogenies (Patchell et al., 2014) with *Polanisia* sister to *Cleome* sensu stricto, but differing in the placement of *Gynandropsi*s (unsupported in Patchell et al., 2014). Additionally, the sampling of only four Capparaceae limited our ability to say much about the relationships within the family; however, to date, there is no phylogeny based solely on nuclear data for this group of plants. Discordance analyses of the Brassicales showed agreement among gene trees for nodes along the backbone, except within and between Clade C, Lineage II, and expanded Lineage II in the Brassicaceae.

### WGD events across the Brassicales

**Figure 2:**
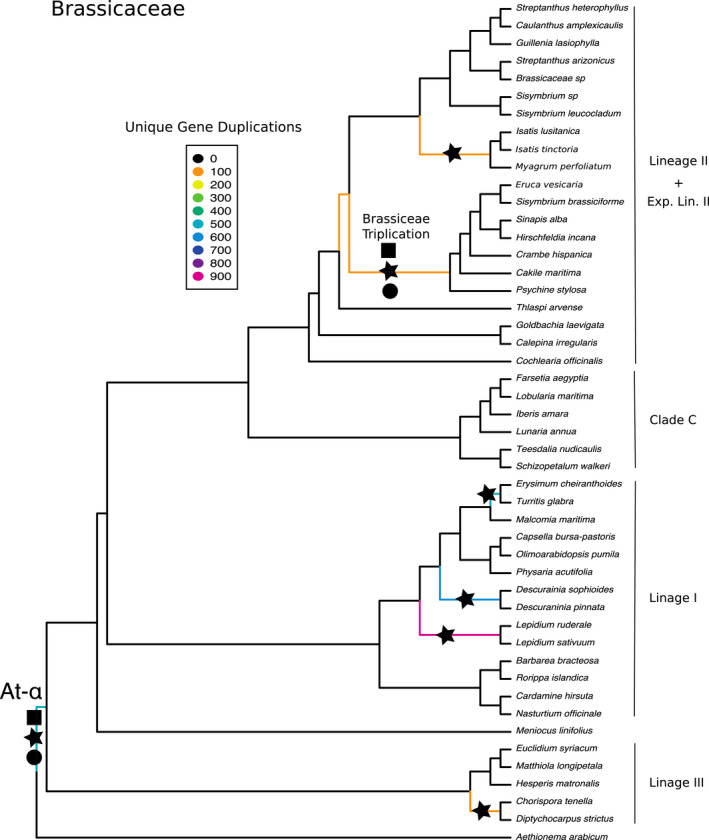
Coalescent‐based species phylogeny and whole‐genome duplication events of the Brassicaceae. Branch color denotes number of unique gene duplications as determined by PUG (github.com/mrmckain/PUG). WGD events identified by PUG (black stars) FASTKs (black squares; McKain et al., 2016); and by DupPipe (black circles; Barker et al., 2010) are indicated. Support values are all >0.7 local posterior probabilities.

Two popular (and most cost‐effective) methods used to detect WGDs across a phylogeny include the analysis of (1) gene tree topologies and (2) *K*_s_ plots, which allowed for the identification of signatures left behind in paralogs after WGD. We used a combination of these approaches to test hypotheses of proposed WGD across the Brassicales. Using PUG (github.com/mrmckain/PUG), a gene tree topology WGD estimation method, we recovered some previously inferred events with high support (e.g., At‐ɑ and At‐β) but did not find strong support for other WGD events, such as the more recent Brassiceae triplication (Fig. 1). Notably, PUG identified only 65 unique gene duplications at the Brassiceae node when only gene trees with >80% bootstrap support were considered. This number is surprisingly low when compared to At‐ɑ and At‐β with counts >300 and >150, respectively. To increase the number of orthogroups used to infer species trees as well as to increase the number of gene trees to query putative paralogs against, we independently analyzed phylogenies of each family for evidence of WGD. By evaluating the Brassicaceae, Capparaceae, Cleomaceae, and [Resedaceae + Bataceae + Moriagaceae + Caricaceae] families separately, we increased gene tree counts in the analyses and improved WGD detection of previously inferred events (Fig. 2).

#### WGD events in Brassicaceae

Analysis of the Brassicaceae identified At‐ɑ at the base of the family and also successfully identified the Brassiceae whole‐genome triplication (Lysak et al., 2005; Tang et al., 2012; Fig. 2). We also recovered neopolyploid (relatively young) events shared between (1) *Chorispora tenella* and *Diptychocarpus strictus*, (2) *Lepidium ruderale* and *L. sativum*, (3) *Descurainia sophioides* and *D. pinnata*, (4) *Turritis glabra* and *Erysimum cheiranthoides*, and (5) a clade of *Isatis lusitanica*, *I. tinctoria*, and *Myagrum perfoliatum*. Using both FASTKs to estimate pairwise *K*_s_ values (github.com/mrmckain/FASTKs; McKain et al., 2016) and DupPipe to estimate *K*_s_ values using duplications in gene trees (Barker et al., 2010), the *K*_s_ plots mostly showed agreement with the WGD events inferred by the phylogenetic method, PUG. For example, *K*_s_ plots from both analyses recovered the Brassiceae triplication (*K*_s_ ~ 0.3; Appendix S9). However, for the neopolyploid events and At‐ɑ, the *K*_s_ plots showed differing results between FASTKs and DupPipe, some with and others without evidence for WGD events (Appendix S9).

#### Independent WGD events in Cleomaceae

**Figure 3:**
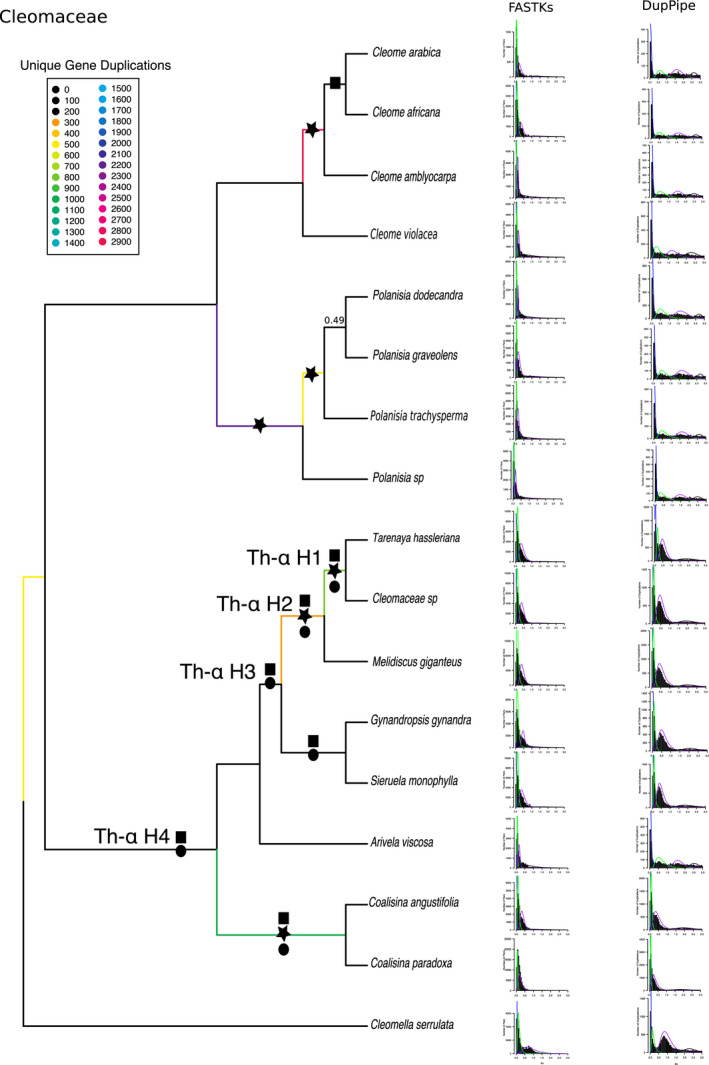
Coalescent‐based species phylogeny and whole‐genome duplication events of the Cleomaceae. Branch color denotes number of unique gene duplications as determined by PUG (github.com/mrmckain/PUG). WGD events identified by PUG (black stars), by FASTKs (black squares; McKain et al., 2016), and by DupPipe (black circles; Barker et al., 2010) are indicated. *K*_s_ plots using both FASTKs and DupPipe are placed next to their corresponding branch. *Y*‐axes of *K*_s_ plots are not congruent: FASTKs measures number of pairs, and DupPipe measures numbers of duplications. Support values are indicated if <0.7 local posterior probability.

When running PUG using the Cleomaceae family, we placed Th‐ɑ as potentially shared between *T. hassleriana* and Cleomaceae sp. We also identified additional events between (1) *Coalisina paradoxa* and *C. angustifolia*; (2) four species of *Polanisia*; and (3) *Cleome amblyocarpa*, *C. africana*, and *C. arabica* (Fig. 3).

Both methods of *K*_s_ estimation provided support for the placement of Th‐ɑ with peaks at ~0.4 for *T. hassleriana* and Cleomaceae sp. and also for *Melidiscus giganteus*, *G. gynandra*, and *Sieruela monophylla*, suggesting that Th‐ɑ is shared across more than just *T. hassleriana* and *Cleomaceae* sp. (Fig. 3). We did not see evidence for this peak in *A. viscosa*, which is sister to the species above. As for the other three events, the story is more complicated. When compared to *C. violacea* (which lacks evidence for Th‐ɑ; Emery et al., 2018; One Thousand Plant Transcriptomes Initiative, 2019), we concluded that there was no evidence for two of these novel events: the event shared by the four species of *Polanisia* and the event shared by *Cleome amblyocarpa*, *C. africana*, and *C. arabica*. However, the event between *Coalisina paradoxa* and *C. angustifolia* does have a signal for a WGD in the *K*_s_ plots (Fig. 3).

**Figure 4:**
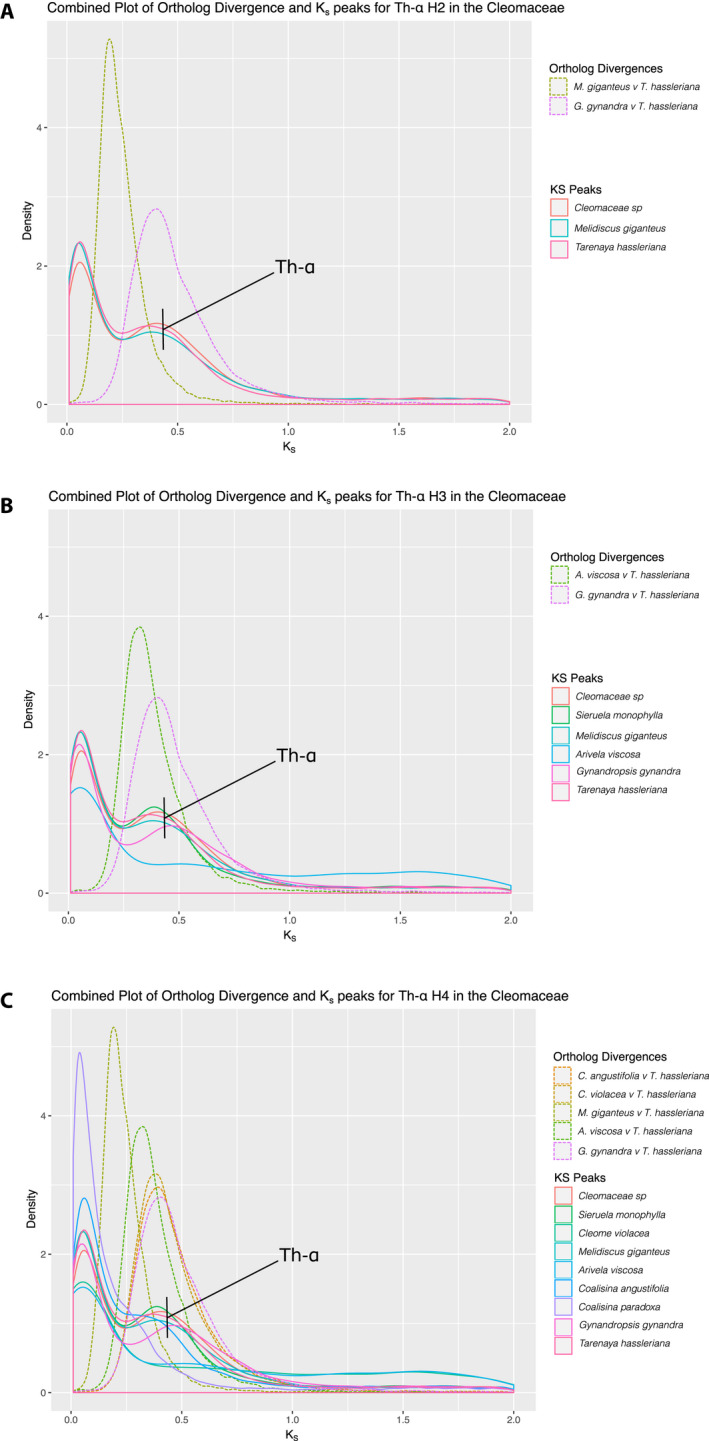
Comparison of ortholog divergences and *K*_s_ peaks of the Cleomaceae to test hypotheses of placement for Th‐ɑ. (A) Testing H2 by comparison of ortholog divergences of *Melidiscus giganteus* and *Gynandropsis gynandra* to *Tarenaya hassleriana* compared with *K*_s_ peaks of Cleomaceae sp., *M. giganteus*, and *T. hassleriana*. (B) Testing of H3 by comparison of ortholog divergence between *Arivela viscosa* and *T. hassleriana*, and *G. gynandra* to *T. hassleriana* with *K*_s_ values of Cleomaceae sp., *Sieruela monophylla*, *M. giganteus*, *A. viscosa*, *G. gynandra*, and *T. hassleriana*. (C) Testing the H4 hypothesis for placement of Th‐ɑ.

Due to incongruence of results for the placement of Th‐ɑ, we divided potential placements into four hypotheses, H1–H4, to test the age of ortholog divergence between taxa to the age of Th‐ɑ (*K*_s_ ~ 0.4). We found evidence that Th‐ɑ is shared with at least *T. hassleriana*, Cleomaceae sp., and *M. giganteus* and that Th‐ɑ occurred before the divergence between *M. giganteus* and *T. hassleriana* and around the same time as the divergence of *G. gynandra* and *T. hassleriana* (Th‐ɑ H2; Fig. 4A). When we compare the divergence between *A. viscosa* and *G. gynandra* to the *K*_s_ values of these three species along with *S. monophylla*, *A. viscosa*, and *G. gynandra*, we found that *A. viscosa* and *G. gynandra* diverged more recently than Th‐ɑ and that, as in earlier *K*_s_ plots, *A. viscosa* lacks evidence for Th‐ɑ (Th‐ɑ H3; Fig. 4B). This perplexing result could indicate that the data from *A. viscosa* is of poor quality or that the genome has lost such a large fraction of the duplicates that the signal for this event is not detected. To further test for the placement of Th‐ɑ, we expanded our comparisons to include the ortholog divergence of *Coalisina angustifolia* and *T. hassleriana* as well as the divergence between *C. violacea* and *T. hassleriana* to test if the proposed independent WGD events between these two clades may be a single event (Th‐ɑ H4; Fig. 4C). Surprisingly, the ortholog divergence for both pairs of taxa is about the same age as Th‐ɑ. Based on these results, Th‐ɑ either is shared across the whole clade (Th‐ɑ H4) or is two separate events that happened at approximately the same time. A comparison of ortholog divergence to *K*_s_ peaks for the two other identified WGD events using phylogenomics suggests that there is no other WGD event in the Cleomaceae (Appendix S10).

#### WGD in Capparaceae

**Figure 5:**
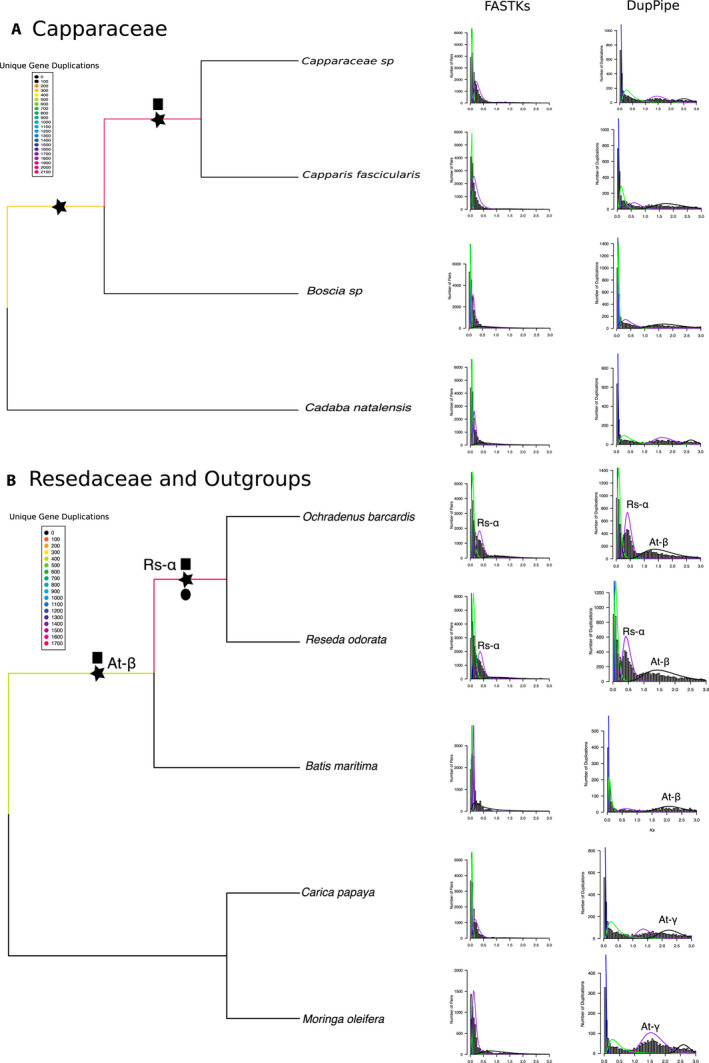
Coalescent‐based species phylogenies and whole‐genome duplication events of the (A) Capparaceae and (B) Resedaceae + Outgroups. Branch color denotes number of unique gene duplications as determined by PUG (github.com/mrmckain/PUG). WGD events identified by PUG (black stars), by FASTKs (black squares; McKain et al., 2016), and by DupPipe (black circles; Barker et al., 2010) are indicated. *K*_s_ plots using both FASTKs and DupPipe are placed next to their corresponding branch. *Y*‐axes of *K*_s_ plots are not congruent: FASTKs measures number of pairs, and DupPipe measures numbers of duplications. At‐β and At‐γ events are noted above corresponding peaks in the *K*_s_ plots. Support values are all >0.7 local posterior probabilities.

In agreement with Lysak (2018), our PUG recovered evidence for an independent WGD event in the Capparaceae that is shared between a species of *Capparis* and another species of Capparaceae included in our analyses (Fig. 5A). This event was supported by *K*_s_ plots using FastKs, but not DupPipe, with a peak centered at *K*_s_ ~ 0.3 (Fig. 5A). Ortholog divergences between members of the Capparaceae showed conflicting patterns. When comparing *K*_s_ values of *Boscia* sp., *Capparis fascicularis*, Capparaceae sp., and *Cadaba natalensis* from DupPipe to the ortholog divergence time between *Boscia* sp. and *Capparis fascicularis*, we found that the divergence between these two species occurs before the possible WGD event, agreeing with the PUG analysis. All four taxa shared a peak in their *K*_s_ distribution, although their *K*_s_ plots from both analyses were not in agreement, providing conflicting results for the identification of this event. The divergences tested between *Boscia* sp. and *Cadaba natalensis* and between *Capparis fascicularis* and *Cadaba natalensis* also occur before the proposed event. However, the divergence between *Capparis fascicularis* and a misidentified species of Capparaceae seems to occur at the same time as the peak in *K*_s_ values (Appendix S11).

#### Resedaceae specific WGD

When combining the Resedaceae (*O. barcardis* and *R. odorata*), Bataceae, Moringaceae, and Caricaceae families together, we found strong evidence for a Resedaceae‐specific WGD event in all three analyses, with *K*_s_ plots indicating a peak at ~0.4 (Fig. 5B). The ortholog divergences seemed to support the proposal of this WGD as shared between the samples of Resedaceae. Both samples (*R. odorata* and *O. barcardis*) shared a *K*_s_ peak of ~0.4, which occurred before the divergence between these two samples and after the divergence between Resedaceae from *B. maritima* (Appendix S11). In addition, we recovered evidence for At‐β using both PUG and DupPipe (*K*_s_ ~ 1.7; Fig. 5B).

## DISCUSSION

Studies of the relationships within the Brassicales have included either many taxa but few genes (Hall et al., 2004; Hall, 2008; Cardinal‐McTeague et al., 2016), a few taxa and few genes (Rodman et al., 1998), or few taxa and many genes (Edger et al., 2015, 2018). In this study, we balance taxa and genes to present a well‐supported chloroplast and nuclear phylogeny for the Brassicales, both in overall agreement with previous studies at the interfamilial and intrafamilial levels (Edger et al., 2015, 2018; Cardinal‐McTeague et al., 2016; Huang et al., 2016; Guo et al., 2017). Using the nuclear phylogeny, we highlight several potential placements of the Th‐ɑ WGD event and identify other possible novel events in the Cleomaceae, Capparaceae, and Resedaceae.

### Incongruences between the chloroplast and nuclear trees across the Brassicaceae

**Figure 6:**
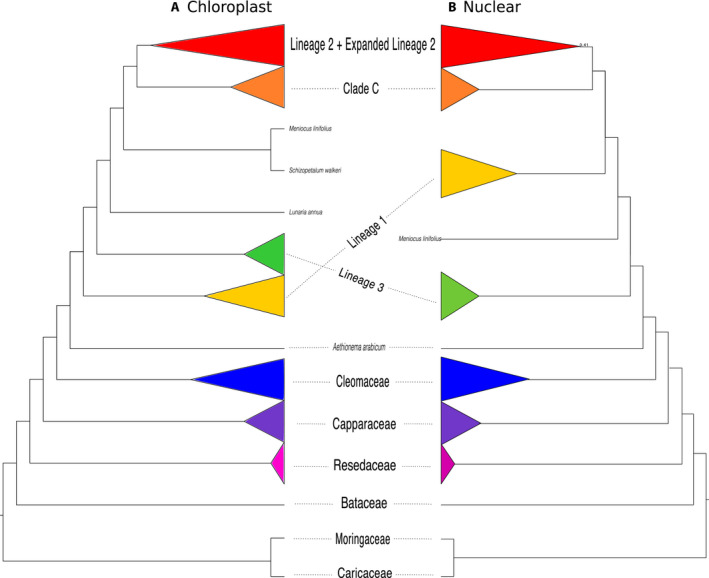
Comparison of (A) maximum likelihood whole‐chloroplast phylogeny to (B) coalescent‐based species phylogeny of the Brassicales. Major lineages and clades of the Brassicaceae are indicated. Support values are indicated if <0.7 local posterior probabilities or <70% bootstrap support.

Although relationships in our nuclear and chloroplast phylogenies are congruent with previous analyses, we highlight incongruence *between* the nuclear and chloroplast trees among the major lineages of the Brassicaceae, a well‐documented pattern between these genomes (Beilstein et al., 2008; Huang et al., 2016; Nikolov et al., 2019; summarized in Fig. 6). We find Lineage I sister to [Lineage III + Lineage II + Expanded Lineage II + Clade C] in the chloroplast tree and Lineage III sister to [Lineage I + Lineage II + Expanded Lineage II + Clade C] in the nuclear tree. Huang et al. (2016), using 113 low‐copy nuclear genes from 55 Brassicaceae species, recovered a tree congruent with our nuclear phylogeny, as did Nikolov et al. (2019) in their study using 79 species and 1421 exons. Additionally, Guo et al. (2017), using 77 chloroplast genes from 53 samples, recovered a phylogeny in agreement with our chloroplast tree. With additional taxon sampling, an increase in data, and using the same samples across analyses, we too recover incongruent relationships between nuclear and chloroplast trees, leaving us to conclude that the trees from these genomes are in disagreement due to different evolutionary histories, which could include ancient hybridization or introgression events (Forsythe et al., 2018 [Preprint]). For future users of these phylogenies, the differences between these two trees are important to consider when using the phylogeny to assess character evolution and divergence dating, as node ordering depends on which tree is used.

### Putative placement of Th‐ɑ in the Cleomaceae

Previous studies have identified a WGD event unique to Cleomaceae (Th‐ɑ) using a variety of sources from syntenic regions to ESTs (Schranz and Mitchell‐Olds, 2006; Barker et al., 2009; reviewed in Bayat et al., 2018; One Thousand Plant Transcriptomes Initiative, 2019). Placement of Th‐ɑ within the Cleomaceae had yet to be confirmed. By using *K*_s_ plots to assess for the signatures of WGD left behind in the paralogs they created, phylogenetics using individual gene tree topologies, gene counts, a known species tree, and ortholog divergences, we are able to putatively place Th‐ɑ as shared between *T. hassleriana*, Cleomaceae sp., *Melidiscus giganteus*, *G. gynandra*, and *S. monophyla*, *A. viscosa*, *Coalisina angustifolia*, and *Coalisina paradoxa* (Th‐ɑ H4; Fig. 3). We include these last three species due to both the evidence from ortholog divergences and the signatures in *K*_s_ plots that strongly suggest this event is shared with all species (Figs. 3 and 4). It is possible that two separate events occurred independently and that *A. viscosa* does indeed lack a WGD. Although this hypothesis (Th‐ɑ H3) is in agreement with the most recent analysis of this group by the One Thousand Plant Transcriptomes Initiative (2019), their sampling of the Cleomaceae was limited and did not include *Coalisina angustifolia* and *C. paradoxa*. *K*_s_ plots of all samples, other than *A. viscosa*, identify a peak at *K*_s_ ~ 0.4, agreeing with previous studies (Barker et al., 2009; van den Bergh et al., 2014) that first identified this peak in *T. hassleriana* followed by *G. gynandra*. PUG, however, supports two separate events. The difficulty in placing this event may be due to the gene‐tree discordance found within this clade (Fig. 1) or to the possibility that, like others in the order, it is a whole‐genome triplication, in which case it will be hard to tease apart due to having to resolve two serial duplication events that occurred in a short period (as with the tribe Brassiceae; Tang et al., 2012).

### Multiple WGD events in the Cleomaceae?

In addition to placing Th‐ɑ, we report two possible additional WGD events in the Cleomaceae. Both events are identified in the Brassicales and the Cleomaceae analyses, but with much greater support in the analysis of the Cleomaceae species. These WGD events are placed at common ancestors shared between (1) *Cleome amblyocarpa*, *C. africana*, and *C. arabica* and (2) four species of *Polanisia* (Fig. 3). *K*_s_ plots provide contrasting support for these events. *K*_s_ plots from FASTKs of *C. africana* and *C. arabica* show a small peak of duplicates at *K*_s_ ~ 0.3. Yet there is no evidence of a WGD event when the same data are run through DupPipe. The *K*_s_ plots of *C. amblyocarpa* also give conflicting evidence for this event. The *K*_s_ plot from FASTKs looks much more similar to that of *C. violacea*, which shows no evidence of a recent WGD event (based on genome sequencing; Emery et al., 2018). The second event shared between the four species of *Polanisia* is supported by a large number (2200) of unique gene duplications using PUG but not by *K*_s_ plots from either FASTKs or DupPipe; the resulting plots look more similar to *C. violacea*. Analyses of ortholog divergence between *C. amblyocarpa*, *C. africana*, and *C. arabica* also lack support for a WGD (Appendix S10), as do analyses between the four species of *Polanisia* (Appendix S10). To further test how WGD and C_4_ photosynthesis have evolved in this family, we suggest a study focusing primarily on Cleomaceae sampling. C_4_ photosynthesis has evolved at least three times independently in Cleomaceae, specifically in (of the taxa sampled) *G. gynandra* and *Coalisina angustifolia* with *C. paradoxa* as a C_3_‐C_4_ intermediate in anatomy and physiology (Bhide et al., 2014). If our putative placement of Th‐ɑ is correct, then all of these samples share this event. It will be interesting to investigate the role of polyploidy and, more specifically, Th‐ɑ, in character evolution in this group.

### WGD events in the Capparaceae and Resedaceae

Although our analysis included only two samples, we recover some support for an event between at least one species of *Capparis* and the misidentified species of Capparaceae (Fig. 5A). Given this ambiguous species identification, inconclusiveness from *K*_s_ plots, and no support in comparison between ortholog divergence and *K*_s_ peaks, this event, although supported by many unique duplicates in the PUG analysis, should be interpreted carefully. That said, Lysak (2018), using chromosome counts, also proposed that Capparaceae experienced a unique event; however, chromosome counts alone may be misleading in concluding that a WGD event has occurred (Evans et al., 2017). There is also a lack of agreement between *K*_s_ plots derived using FASTKs and DupPipe, which estimate *K*_s_ values differently (i.e., pairwise *K*_s_ estimates in FASTKs vs. estimates of *K*_s_ at nodes in gene trees in DupPipe), further confounding evidence for either presence or absence of a Capparaceae‐specific event. Between information presented by Lysak (2018) and the evidence presented here, this possible event certainly warrants additional study.

We find good evidence to support the presence of a separate WGD event in the Resedaceae, also hypothesized by Lysak (2018). This polyploidy is one of the few events recovered with consensus between *K*_s_ plots (from both FASTKs and DupPipe), phylogenetics, and ortholog divergences (Fig. 4B and Appendix S11). Therefore, we are confident in the identification of this event (named Rs‐ɑ here). This event was recently identified using a single species (*Reseda odorata*; One Thousand Plant Transcriptomes Initiative, 2019); however, we determine that it is shared with at least one other species in the family, *Ochradenus barcardis*. The sister families, Caricaceae and Moringaceae, show no evidence of unique WGD events, which is in agreement with the recent whole‐genome sequencing of *Moringa oleifera* (Chang et al., 2018). When Tian et al. (2015) compared the papaya genome, which shows no evidence of a (recent) WGD (Ming et al., 2008), to the newly sequenced genome of *M. oleifera*, they too concluded that Moringaceae did not experience a family‐specific genome duplication. The identified and well‐supported Resedaceae event warrants additional sampling and investigation to test if it is shared across the whole family.

### Methodological challenges with placing WGD events: sampling matters

Currently, three types of methods are used to detect WGD events: (1) *K*_s_ plots to assess for signatures left behind in paralogs after WGD, (2) identification of retained duplicate blocks in a genome, and (3) phylogenetics using individual gene‐tree topologies. Since all three methods have limitations in identifying WGD events, we use a combination of approaches to test hypotheses, reduce proposing events that may not exist, and simultaneously provide multiple lines of evidence for recovered events.

Recently, an abundance of papers have highlighted the difficulties and complexities of determining WGD events across the tree of life (Conover et al., 2018; Tiley et al., 2018; Li and Barker, 2019 [Preprint]; Li et al., 2019; Nakatani and McLysaght, 2019; Zwaenepoel and van de Peer, 2019 [Preprint]; Zwaenepoel et al., 2019). We add another dimension to this conversation by demonstrating that the different taxonomic levels from which we sample, such as the order or family, make a difference in support of previously identified WGDs (i.e., the Brassiceae triplication). Recent research has demonstrated that differences in taxonomic sampling and taxon occupancy in data matrices can influence the inference of WGDs, particularly if adding taxa decreases taxon occupancy in gene families (Yang et al., 2015; Li et al., 2018; Li and Barker, 2019 [Preprint]; Zwaenepoel and van de Peer, 2019 [Preprint]). Testing for WGD events across the Brassicales phylogeny led to less certain topologies; therefore, signals of WGD are missed when filtering for nodes with only high bootstrap support to count duplicates. To account for this and to increase taxon and gene‐family occupancy in our data sets, we reduce sampling to just the family level to infer WGD. However, at each level of analysis, we choose an arbitrary cutoff for the number of duplicates that we feel is sufficient to infer a WGD event, a documented criticism of these types of methods (Zwaenepoel and van de Peer, 2019). Others note that it is also important to consider heterogeneity in substitution rates (Cui et al., 2006; Barker et al., 2009; Yang et al., 2015), but phylogenomic methods and denser sampling should obviate the need for rate corrections. Variation in the duplication and loss rate across the species tree may also impact tests for WGD events (Li et al., 2018; Zwaenepoel and van de Peer, 2019 [Preprint]).

Although our *K*_s_‐based inferences of WGDs are largely consistent with phylogenomic inferences, there are some differences among the approaches. FASTKs and DupPipe use different estimates of *K*_s_ that likely produce the observable differences in the respective *K*_s_ plots. FASTKs uses a pairwise approach for estimating *K*_s_ values (github.com/mrmckain/FASTKs; McKain et al., 2016), whereas DupPipe estimates *K*_s_ values from nodes of gene trees (Barker et al., 2010). Tiley et al. (2018) explored the difference in *K*_s_ estimates from these types of approaches and found that the observed differences in peaks of duplications between the two different methods is consistent with simulations. The node‐based estimates of *K*_s_ from DupPipe often yield sharper peaks in putative WGDs, with overall lower numbers of duplications because of the difference in number of nodes vs. pairwise comparisons. However, the results of both approaches are largely consistent after close inspection. Perhaps more confounding for *K*_s_ analyses is the interpretation of mixture models to identify putative peaks associated with a WGD. Mixture models, which are typically fit to the distribution of duplicates, tend to overestimate the number of true peaks (Naik et al., 2007; Tiley et al., 2018; Zwaenepoel et al., 2019). Using two different methods across multiple species allowed us to evaluate and compare putative peaks from different analyses to identify the expected signatures of WGDs. Furthermore, because paralogs from WGDs tend to be more highly expressed than those resulting from tandem duplications (Casneuf et al., 2006), transcriptomes may yield a data set that is more enriched for WGD duplicates than (fragmented) genomic data. Therefore, transcriptome data, as shown by Tiley et al. (2018), may actually improve success in detecting WGD events.

## CONCLUSIONS

The Brassicales is an excellent group for comparing methods of WGD identification because of the wealth of genomic data and the previously inferred WGDs that are available. With many chromosome‐level genomes available, analyses based on synteny, which seem to be regarded as most reliable in detecting these events (Nakatani and McLysaght, 2019), can be used as controls for comparing WGD methods. Sequenced genomes, which are placed throughout the Brassicales, provide strong evidence for taxa that we know do not have recent WGD events (i.e., *Cleome violacea* and *Carica papaya*) and for taxa that do show evidence for recent WGD events (i.e., *A. thaliana* and many *Brassica* crops). These resources provide calibration points that can be used to verify results when testing for novel events. This group of plants, combined with recent insights on difficulties in placing WGD events, can help further the development of innovative methods in describing and identifying WGDs.

## AUTHOR CONTRIBUTIONS

M.E.M., J.C.P., G.C.C., J.C.H., P.P.E., and M.E.S. designed the project. M.E.M., J.M.B., H.A., J.D.W., and W.T.D. grew plants, collected tissue, and isolated RNA. M.E.M. and J.M.B. isolated DNA. J.C.H. contributed *Cleomella serrulate*. M.E.M. and J.M.B. analyzed the data. P.D.B. and J.D.W. assisted with processing and analyzing the data. C.A.B. wrote alignment filtering scripts. M.S.B. and B.S. analyzed data using DupPipe and OrthoPipe. M.R.M. helped with processing data using FastPlast. I.A. and J.C.H. updated nomenclature and helped correct plant identifications. M.E.M. wrote the original manuscript draft, which was read and approved by all authors.

## Supporting information


**APPENDIX S1.** Current understanding of the phylogenetic relationships between the 17 families of the Brassicales and whole‐genome duplication events.

**APPENDIX S2.** Taxon sampling, accessions, and additional analysis information.

**APPENDIX S3.** RNA and DNA extraction method, library preparation method, sequencing method, read size, and raw read numbers.

**APPENDIX S4.** Orthogroups retained for each analysis.

**APPENDIX S5.** BIC scores for 1–4 components for both FASTKs (McKain et al., 2016) and DupPipe (Barker et al., 2010).

**APPENDIX S6.** BUSCO analysis of de novo transcriptomes.

**APPENDIX S7.** Maximum likelihood phylogeny of the Brassicales using two chloroplast genes, *MatK* and *NdhF*.

**APPENDIX S8.** Maximum likelihood whole chloroplast phylogeny of the Brassicales.

**APPENDIX S9.** Brassicaceae *K*_s_ plots using both FASTKs (McKain et al., 2016) and DupPipe (Barker et al., 2010).

**APPENDIX S10.** Additional ortholog divergences and *K*_s_ peaks of the Cleomaceae.

**APPENDIX S11.** Ortholog divergences and *K*_s_ peaks of the (A) Capparaceae and (B) Resedaceae + Outgroups.

## Data Availability

Sequence data from this article can be found in the NCBI SRA data libraries under BioProject accession number PRJNA542714. Individual BioSample accession numbers can be found in Appendix S2. Seeds corresponding to samples are available upon request.
