# Supplementary material for: Phylogeny and multiple independent whole‐genome duplication events in the Brassicales
Source: Am J Bot. 2020 Aug 24;107(8):1148–64. doi: 10.1002/ajb2.1514 (PMC7496422; doi:10.1002/ajb2.1514)
Supplement: Supplementary file 1 — APPENDIX S1. Current understanding of the phylogenetic relationships between the 17 families of the Brassicales and whole‐genome duplication events. [file AJB2-107-1148-s001.pdf]

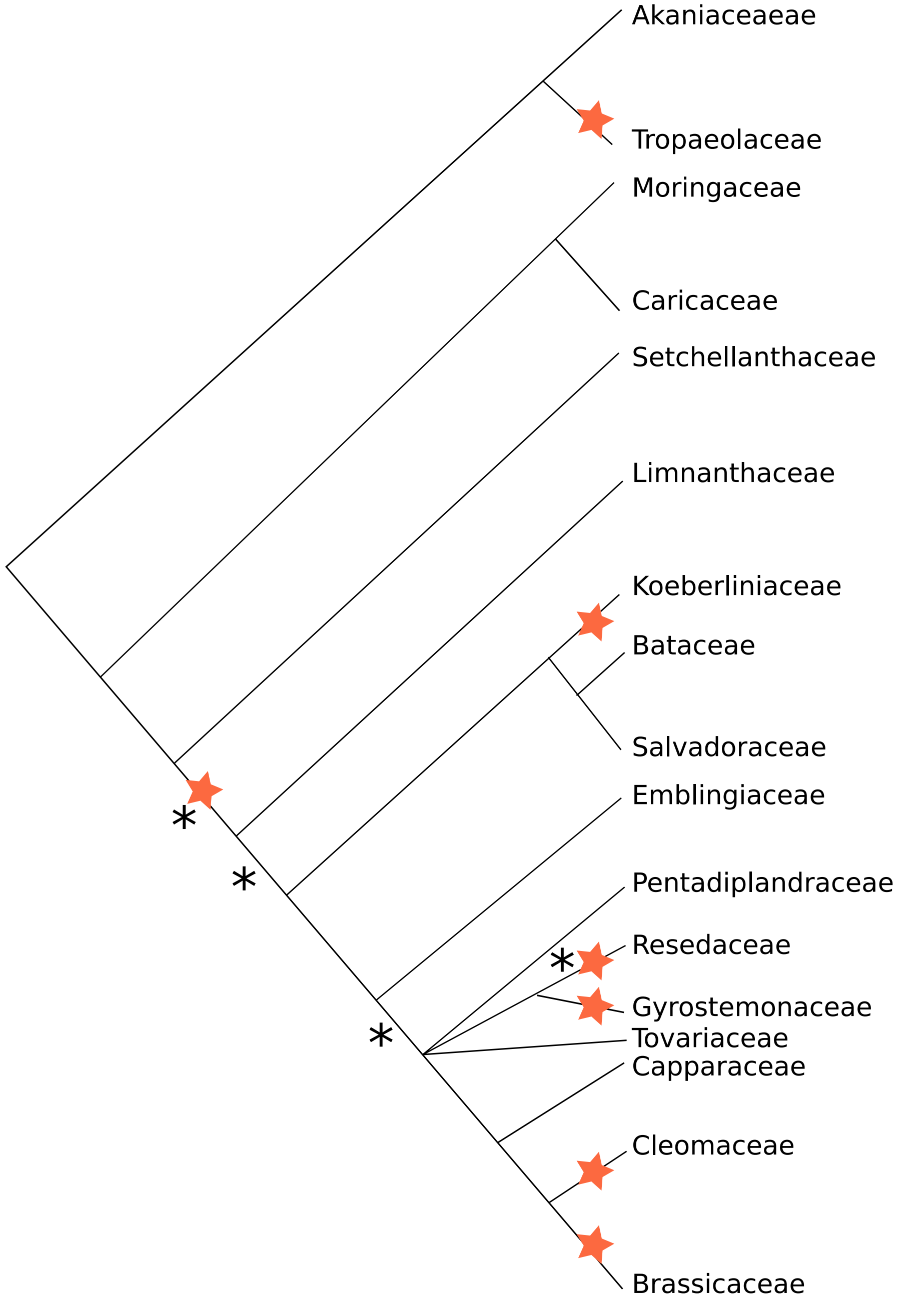

Appendix S1. Current understanding of the relationships between the 17 families of the Brassicales (APG IV). An asterisk (*) indicates branch support between 50-80%. All other branches have greater than 80% support. Orange stars indicate whole-genome duplication events supported by the One Thousand Plant Transcriptomes Initiative (2019). It is uncertain if the At-β event is shared by Setchellanthaceae or not (latter scenario is indicated; but see Edger et al. 2018
