# Supplementary material for: Phylogeny and multiple independent whole‐genome duplication events in the Brassicales
Source: Am J Bot. 2020 Aug 24;107(8):1148–64. doi: 10.1002/ajb2.1514 (PMC7496422; doi:10.1002/ajb2.1514)
Supplement: Supplementary file 2 — APPENDIX S2. Taxon sampling, accessions, and additional analysis information. [file AJB2-107-1148-s002.pdf]

Appendix S2. Taxon sampling with authorship (determined using http://www.ipni.org/index.html), seed accessions and the collections they are from, additional analysis information, and SRA accessions. SSC = small single copy, LSC= large single copy, IR = inverted repeat.

Family	Genus	Species and Authorship	Seed Accession	Collection	Transcriptome	Chloroplast SSC	Chloroplast LSC	Chloroplast IR	Fast-Plast Subsample	SRA Accession
Bataceae	*Batis*	*maritima* L.	*NA*	Pat Edger	X	X	X	X	3500000	SAMN12815478
Brassicaceae	*Aethionema*	*arabicum* (L.) Andrz. ex DC.	1010	Eric Schranz	X	X	X	X		SAMN12815475
Brassicaceae	*Barbarea*	*bracteosa* Guss.	6203-84	Gomez Campo	X	X	X	X		SAMN12815477
Brassicaceae	Brassicaceae Burnett	sp.	Colorado diploid	Chris Pires	X	X	X	X		SAMN12815542
Brassicaceae	*Cakile*	*maritima* Scop.	B2005-875	Marcus Koch	X	X	X	X		SAMN12815481
Brassicaceae	*Calepina*	*irregularis* Thell.	2158-72	Gomez Campo	X	X	X	X		SAMN12815482
Brassicaceae	*Capsella*	*bursa-pastoris* (L.) Medik.	2006-956	Marcus Koch	X	X	X	X		SAMN12815485
Brassicaceae	*Cardamine*	*hirsuta* L.	2005-701	Marcus Koch	X	X				SAMN12815486
Brassicaceae	*Caulanthus*	*amplexicaulis* S. Watson	*NA*	Alan Pepper	X	X	X	X		SAMN12815488
Brassicaceae	*Chorispora*	*tenella* DC.	2006-629	Marcus Koch	X	X	X	X	1000000	SAMN12815489
Brassicaceae	*Cochlearia*	*officinalis* L.	1910-84	Gomez Campo	X	X	X	X		SAMN12815502
Brassicaceae	*Crambe*	*hispanica* L.	PI388853	USDA	X	X	X	X		SAMN12815503
Brassicaceae	*Descurainia*	*pinnata* (Walter) Britton	B2004-26	Marcus Koch	X	X	X	X		SAMN12815505
Brassicaceae	*Descurainia*	*sophioides* (Fisch.) O.E. Schulz	BarbGH	Chris Pires	X	X	X	X		SAMN12815504
Brassicaceae	*Diptycocarpus*	*strictus* (Fisch. ex M.Bieb.) Trautv	05-0397-10-00	Klaus Mummenhoff	X					SAMN12815506
Brassicaceae	*Eruca*	*vesicaria* (L.) Cav.	PI633218	USDA	X	X	X	X	1000000	SAMN12815507
Brassicaceae	*Erysimum*	*cheiranthoides* L.	03-0030-10-00	Klaus Mummenhoff	X	X	X	X		SAMN12815508
Brassicaceae	*Euclidium*	*syriacum* (L.) W.T. Aiton	0587-68	Gomez Campo	X	X	X	X		SAMN12815509
Brassicaceae	*Farsetia*	*aegyptia* Turra	96-001-10-00	Klaus Mummenhoff	X		X			SAMN12815510
Brassicaceae	*Goldbachia*	*laevigata* DC.	*NA*	Martin Lysak	X	X	X	X		SAMN12815511
Brassicaceae	*Guillenia*	*lasiophylla* Green	Costal type	Chris Pires	X	X	X	X		SAMN12815512
Brassicaceae	*Hesperis*	*matronalis* L.	0039-68	Gomez Campo	X	X	X	X	1000000	SAMN12815514
Brassicaceae	*Hirschfeldia*	*incana* (L.) Lagr.-Foss	2024-71	Gomez Campo	X	X	X	X		SAMN12815515

Brassicaceae	*Iberis*	*amara* L.	2005-238	Marcus Koch	X	X	X	X		SAMN12815516
Brassicaceae	*Isatis*	*lusitanica* L.	01-0035-10-00	Klaus Mummenhoff	X	X	X	X		SAMN12815517
Brassicaceae	*Isatis*	*tinctorial* L.	B2009-0378	Marcus Koch	X	X	X	X		SAMN12815518
Brassicaceae	*Leavenworthia*	*uniflora* (Michx.) Britton	2004-557	Marcus Koch		X	X	X		SAMN12815549
Brassicaceae	*Lepidium*	*ruderale* L.	05-0190-10-00	Klaus Mummenhoff	X	X	X	X		SAMN12815519
Brassicaceae	*Lepidium*	*sativum* L.	2003-169	Marcus Koch	X	X	X	X		SAMN12815520
Brassicaceae	*Lobularia*	*maritima* (L.) Desv.	B2009-0390	Marcus Koch	X	X	X	X		SAMN12815521
Brassicaceae	*Lunaria*	*annua* L.	2006-705	Marcus Koch	X	X	X	X		SAMN12815522
Brassicaceae	*Malcolmia*	*maritima* (L.) W.T. Aiton	2008-777	Marcus Koch	X	X	X	X	2500000	SAMN12815523
Brassicaceae	*Matthiola*	*longipetala* DC.	1351-70	Gomez Campo	X	X	X	X		SAMN12815524
Brassicaceae	*Meniocus*	*linifolius* (Willd.) DC.	0893-68	Gomez Campo	X	X	X	X	1000000	SAMN12815476
Brassicaceae	*Myagrum*	*perfoliatum* L.	2006-432	Marcus Koch	X	X	X	X		SAMN12815526
Brassicaceae	*Nasturtium*	*officinale* W.T.Aiton	AMC7.20.15	Chris Pires	X	X	X	X		SAMN12815527
Brassicaceae	*Olimarabidopsis*	*pumila* (Stephan) Al-Shehbaz, O'Kane & R.A.Price	BarbGH	Chris Pires	X					SAMN12815529
Brassicaceae	*Physaria*	*acutifolia* Rydb.	01201.13 ALPLAINS 50	Chris Pires	X	X	X	X		SAMN12815530
Brassicaceae	*Psychine*	*stylosa* Desf.	B2004-78	Marcus Koch	X	X	X	X		SAMN12815535
Brassicaceae	*Rorippa*	*islandica* (Oeder) Borbás	2006-89	Gomez Campo	X	X	X	X		SAMN12815537
Brassicaceae	*Schizopetalum*	*walkeri* Sims	*NA*	Martin Lysak	X	X	X	X		SAMN12815538
Brassicaceae	*Sinapis*	*alba* L.	0560-79	Gomez Campo	X	X	X	X	1000000	SAMN12815539
Brassicaceae	*Sisymbrium*	*brassiciforme* C.A.Mey	04-0372-10-00	Klaus Mummenhoff	X	X	X	X		SAMN12815540
Brassicaceae	*Sisymbrium*	*leucocladum* (Boiss.) D.A.German & Al-Shehbaz	3760-75	Gomez Campo	X	X	X	X		SAMN12815534
Brassicaceae	*Sisymbrium* L.	sp.	2004-557	Marcus Koch	X					SAMN12815541
Brassicaceae	*Streptanthus*	*arizonicus* S.Watson	02-0095-00-00	Klaus Mummenhoff	X	X	X	X		SAMN12815543
Brassicaceae	*Streptanthus*	*heterophyllus* Nutt.	1730-71	Gomez Campo	X	X	X	X		SAMN12815544
Brassicaceae	*Teesdalia*	*nudicaulis* (L.) W.T.Aiton	2219-73	Gomez Campo	X	X	X	X		SAMN12815546
Brassicaceae	*Thlaspi*	*arvense* L.	1211-67	Gomez Campo	X	X	X	X		SAMN12815547
Brassicaceae	*Turritis*	*glabra* L.	04-0314-10-00	Klaus Mummenhoff	X	X	X	X	4500000	SAMN12815548

Capparaceae	*Boscia* Lam.	sp.	SHN 12-075	Silverhill seeds and books	X	X	X	X		SAMN12815479
Capparaceae	*Cadaba*	*natalensis* Sond.	SHN 12-076	Silverhill seeds and books	X	X	X	X		SAMN12815480
Capparaceae	Capparaceae Juss.	sp.	SHN 12-079	Silverhill seeds and books	X	X	X	X		SAMN12815484
Capparaceae	*Capparis*	*fascicularis* DC.	SHN 12-078	Silverhill seeds and books	X	X	X	X		SAMN12815483
Cariaceae	*Carica*	*papaya* L.	*NA*	Chris Pires	X	X	X	X		SAMN12815487
Cleomaceae	*Arivela*	*viscosa* (L.) Raf.	KEW 104126	Kew Gardens	X	X	X	X		SAMN12815500
Cleomaceae	Cleomaceae Horan.	sp.	KEW 36588	Kew Gardens	X	X	X	X		SAMN12815497
Cleomaceae	*Cleome*	*africana* Botsch.	*NA*	Chris Pires	X	X	X	X		SAMN12815490
Cleomaceae	*Cleome*	*amblyocarpa* Barratte & Murb.	151485	Jocelyn Hall	X					SAMN12815491
Cleomaceae	*Cleome*	*arabica* L.	*NA*	Jocelyn Hall	X	X	X	X		SAMN12815493
Cleomaceae	*Cleome*	*violacea* L.	*NA*	Jocelyn Hall	X	X	X	X		SAMN12815498
Cleomaceae	*Cleomella*	*serrulata* (Pursh) Roalson & J.C.Hall	*NA*	Jocelyn Hall	X					SAMN12815501
Cleomaceae	*Coalisina*	*angustifolia* Raf.	KEW 82633	Kew Gardens	X	X	X	X	2000000	SAMN12815492
Cleomaceae	*Coalisina*	*paradoxa* (R.Br. ex DC.) Roalson & J.C.Hall	KEW 109051	Kew Gardens	X	X	X	X	1000000	SAMN12815496
Cleomaceae	*Gynandropsis*	*gynandra* (L.) Briq.	*NA*	Jocelyn Hall	X	X	X	X		SAMN12815513
Cleomaceae	*Melidiscus*	*giganteus* Raf.	*NA*	Jocelyn Hall	X	X	X	X		SAMN12815499
Cleomaceae	*Polanisia*	*dodecandra* (L.) DC.	2006-372	Marcus Koch	X	X				SAMN12815531
Cleomaceae	*Polanisia*	*graveolens* Raf.	2004 -157	Marcus Koch	X	X	X	X		SAMN12815532
Cleomaceae	*Polanisia* Raf.	sp.	KEW 93745	Kew Gardens	X	X	X	X		SAMN12815494
Cleomaceae	*Polanisia*	*trachysperma* Torr. & A.Gray	2006-365	Marcus Koch	X	X	X	X	1600000	SAMN12815533
Cleomaceae	*Sieruela*	*monophylla* (L.) Roalson & J.C.Hall	KEW 413163	Kew Gardens	X					SAMN12815495
Cleomaceae	*Tarenaya*	*hassleriana* (Chodat) Iltis	KEW 48516	Kew Gardens	X	X	X	X		SAMN12815545
Moringaceae	*Moringa*	*oleifera* Lam.	*NA*	Chris Pires	X	X	X	X	1000000	SAMN12815525
Resadaceae	*Ochradenus*	*baccatus* Delile	DMJ0324	Chris Pires	X	X	X	X		SAMN12815528
Resadaceae	*Reseda*	*ordorata* L.	*NA*	Larry Chandler	X	X	X	X	1000000	SAMN12815536
