# Supplementary material for: Phylogeny and multiple independent whole‐genome duplication events in the Brassicales
Source: Am J Bot. 2020 Aug 24;107(8):1148–64. doi: 10.1002/ajb2.1514 (PMC7496422; doi:10.1002/ajb2.1514)
Supplement: Supplementary file 3 — APPENDIX S3. RNA and DNA extraction method, library preparation method, sequencing method, read size, and raw read numbers. [file AJB2-107-1148-s003.pdf]

Appendix S3. RNA and DNA extraction method, library preparation method, sequencing method, read size, and raw read numbers.

Family	Genus	Species	RNA Extraction Kit	RNA Library Prep	RNA Seq Machine	RNA Read Length	RNA Raw Reads	DNA Extraction Kit	DNA Library Prep	DNA Seq Machine	DNA Read Length	DNA Raw Reads
Bataceae	*Batis*	*maritima*	ThermoFisher PureLink RNA Mini Kit	TruSeq	NextSeq	2X75	16565629	Qiagen DNeasy	TruSeq	NextSeq	2X150	8309055
Brassicaceae	*Aethionema*	*arabicum*	ThermoFisher PureLink RNA Mini Kit	TruSeq	NextSeq	2X75	55433848	Qiagen DNeasy	TruSeq	NextSeq	2X150	8757856
Brassicaceae	*Barbarea*	*bracteosa*	ThermoFisher PureLink RNA Mini Kit	TruSeq	NextSeq	2X75	53269435	Qiagen DNeasy	TruSeq	NextSeq	2X150	8979330
Brassicaceae	Brassicaceae	sp.	ThermoFisher PureLink RNA Mini Kit	TruSeq	HiSeq	2X100	40618374	Qiagen DNeasy	TruSeq	NextSeq	2X150	7179797
Brassicaceae	*Cakile*	*maritima*	ThermoFisher PureLink RNA Mini Kit	TruSeq	HiSeq	2X250	5555024	Qiagen DNeasy	TruSeq	NextSeq	2X150	10054961
Brassicaceae	*Calepina*	*irregularis*	ThermoFisher PureLink RNA Mini Kit	TruSeq	NextSeq	2X75	16367748	Qiagen DNeasy	TruSeq	NextSeq	2X150	9472593
Brassicaceae	*Capsella*	*bursa-pastoris*	ThermoFisher PureLink RNA Mini Kit	TruSeq	NextSeq	2X75	23770081	Qiagen DNeasy	TruSeq	NextSeq	2X150	9962200
Brassicaceae	*Cardamine*	*hirsuta*	ThermoFisher PureLink RNA Mini Kit	TruSeq	NextSeq	2X75	23276338	Qiagen DNeasy	TruSeq	NextSeq	2X150	7163619
Brassicaceae	*Caulanthus*	*amplexicaulis*	ThermoFisher PureLink RNA Mini Kit	TruSeq	HiSeq	2X100	30561937	Qiagen DNeasy	TruSeq	NextSeq	2X150	8638165
Brassicaceae	*Chorispora*	*tenella*	ThermoFisher PureLink RNA Mini Kit	TruSeq	NextSeq	2X75	19065280	Qiagen DNeasy	TruSeq	NextSeq	2X150	10713329
Brassicaceae	*Cochlearia*	*officinalis*	ThermoFisher PureLink RNA Mini Kit	TruSeq	NextSeq	2X75	19278016	Qiagen DNeasy	TruSeq	NextSeq	2X150	9785784
Brassicaceae	*Crambe*	*hispanica*	ThermoFisher PureLink RNA Mini Kit	TruSeq	HiSeq	2X100	6677521	Qiagen DNeasy	TruSeq	NextSeq	2X150	8981402
Brassicaceae	*Descurainia*	*pinnata*	ThermoFisher PureLink RNA Mini Kit	TruSeq	HiSeq	2X100	8315473	Qiagen DNeasy	TruSeq	NextSeq	2X150	10532085
Brassicaceae	*Descurainia*	*sophioides*	ThermoFisher PureLink RNA Mini Kit	TruSeq	HiSeq	2X100	41235763	Qiagen DNeasy	TruSeq	NextSeq	2X150	7149869
Brassicaceae	*Diptycocarpus*	*strictus*	ThermoFisher PureLink RNA Mini Kit	TruSeq	HiSeq	2X100	7422199					7488205
Brassicaceae	*Eruca*	*vesicaria*	ThermoFisher PureLink RNA Mini Kit	TruSeq	HiSeq	2X100	7743240	Qiagen DNeasy	TruSeq	NextSeq	2X150	9696734
Brassicaceae	*Erysimum*	*cheiranthoides*	ThermoFisher PureLink RNA Mini Kit	TruSeq	NextSeq	2X75	19481894	Qiagen DNeasy	TruSeq	NextSeq	2X150	9627950
Brassicaceae	*Euclidium*	*syriacum*	ThermoFisher PureLink RNA Mini Kit	TruSeq	HiSeq	2X100	7871790	Qiagen DNeasy	TruSeq	NextSeq	2X150	8499185
Brassicaceae	*Farsetia*	*aegyptia*	ThermoFisher PureLink RNA Mini Kit	TruSeq	NextSeq	2X75	49764414	Qiagen DNeasy	TruSeq	NextSeq	2X150	8514984
Brassicaceae	*Goldbachia*	*laevigata*	ThermoFisher PureLink RNA Mini Kit	TruSeq	NextSeq	2X75	16809960	Qiagen DNeasy	TruSeq	NextSeq	2X150	9849790
Brassicaceae	*Guillenia*	*lasiophylla*	ThermoFisher PureLink RNA Mini Kit	TruSeq	NextSeq	2X75	23503171	Qiagen DNeasy	TruSeq	NextSeq	2X150	10824454
Brassicaceae	*Hesperis*	*matronalis*	ThermoFisher PureLink RNA Mini Kit	TruSeq	NextSeq	2X75	51877498	Qiagen DNeasy	TruSeq	NextSeq	2X150	7807320
Brassicaceae	*Hirschfeldia*	*incana*	ThermoFisher PureLink RNA Mini Kit	TruSeq	NextSeq	2X75	18013197	Qiagen DNeasy	TruSeq	NextSeq	2X150	11527537
Brassicaceae	*Iberis*	*amara*	ThermoFisher PureLink RNA Mini Kit	TruSeq	HiSeq	2X100	7587536	Qiagen DNeasy	TruSeq	NextSeq	2X150	6845991
Brassicaceae	*Isatis*	*lusitanica*	ThermoFisher PureLink RNA Mini Kit	TruSeq	NextSeq	2X75	22464165	Qiagen DNeasy	TruSeq	NextSeq	2X150	11695349
Brassicaceae	*Isatis*	*tinctoria*	Qiagen RNEasy	TruSeq	HiSeq	2X100	20649046	Qiagen DNeasy	TruSeq	NextSeq	2X150	9446020

Brassicaceae	*Leavenworthia*	*uniflora*	NA	NA	NA	NA	NA	Qiagen DNeasy	TruSeq	NextSeq	2X150	9120176
Brassicaceae	*Lepidium*	*ruderale*	ThermoFisher PureLink RNA Mini Kit	TruSeq	NextSeq	2X75	18623760	Qiagen DNeasy	TruSeq	NextSeq	2X150	9459705
Brassicaceae	*Lepidium*	*sativum*	ThermoFisher PureLink RNA Mini Kit	TruSeq	HiSeq	2X100	7083621	Qiagen DNeasy	TruSeq	NextSeq	2X150	9451177
Brassicaceae	*Lobularia*	*maritima*	ThermoFisher PureLink RNA Mini Kit	TruSeq	NextSeq	2X75	21143830	Qiagen DNeasy	TruSeq	NextSeq	2X150	9578197
Brassicaceae	*Lunaria*	*annua*	ThermoFisher PureLink RNA Mini Kit	TruSeq	HiSeq	2X100	6184646	Qiagen DNeasy	TruSeq	NextSeq	2X150	7316600
Brassicaceae	*Malcolmia*	*maritima*	ThermoFisher PureLink RNA Mini Kit	TruSeq	HiSeq	2X100	7408880	Qiagen DNeasy	TruSeq	NextSeq	2X150	7908019
Brassicaceae	*Matthiola*	*longipetala*	ThermoFisher PureLink RNA Mini Kit	TruSeq	NextSeq	2X75	18984562	Qiagen DNeasy	TruSeq	NextSeq	2X150	10044883
Brassicaceae	*Meniocus*	*linifolius*	ThermoFisher PureLink RNA Mini Kit	TruSeq	NextSeq	2X75	51075472	Qiagen DNeasy	TruSeq	NextSeq	2X150	9512838
Brassicaceae	*Myagrum*	*perfoliatum*	ThermoFisher PureLink RNA Mini Kit	TruSeq	HiSeq	2X100	6922905	Qiagen DNeasy	TruSeq	NextSeq	2X150	6637717
Brassicaceae	*Nasturtium*	*officinale*	ThermoFisher PureLink RNA Mini Kit	TruSeq	NextSeq	2X75	52146124	Qiagen DNeasy	TruSeq	NextSeq	2X150	8184647
Brassicaceae	*Olimarabidopsis*	*pumila*	ThermoFisher PureLink RNA Mini Kit	TruSeq	NextSeq	2X75	19934340	*NA*	*NA*	*NA*	*NA*	*NA*
Brassicaceae	*Physaria*	*acutifolia*	ThermoFisher PureLink RNA Mini Kit	TruSeq	NextSeq	2X75	21947053	Qiagen DNeasy	TruSeq	NextSeq	2X150	9285650
Brassicaceae	*Psychine*	*stylosa*	ThermoFisher PureLink RNA Mini Kit	TruSeq	NextSeq	2X75	17163612	Qiagen DNeasy	TruSeq	NextSeq	2X150	9336172
Brassicaceae	*Rorippa*	*islandica*	ThermoFisher PureLink RNA Mini Kit	TruSeq	HiSeq	2X100	7662263	Qiagen DNeasy	TruSeq	NextSeq	2X150	7161563
Brassicaceae	*Schizopetalum*	*walkeri*	ThermoFisher PureLink RNA Mini Kit	TruSeq	NextSeq	2X75	16524348	Qiagen DNeasy	TruSeq	NextSeq	2X150	9736577
Brassicaceae	*Sinapis*	*alba*	ThermoFisher PureLink RNA Mini Kit	TruSeq	HiSeq	2X100	7602469	Qiagen DNeasy	TruSeq	NextSeq	2X150	9902350
Brassicaceae	*Sisymbrium*	*brassiciforme*	ThermoFisher PureLink RNA Mini Kit	TruSeq	NextSeq	2X75	22582931	Qiagen DNeasy	TruSeq	NextSeq	2X150	9078512
Brassicaceae	*Sisymbrium*	*leucocladum*	ThermoFisher PureLink RNA Mini Kit	TruSeq	NextSeq	2X75	15845037	Qiagen DNeasy	TruSeq	NextSeq	2X150	10653471
Brassicaceae	*Sisymbrium*	sp.	ThermoFisher PureLink RNA Mini Kit	TruSeq	NextSeq	2X75	16438394	*NA*	*NA*	*NA*	*NA*	*NA*
Brassicaceae	*Strepthanthus*	*arizonicus*	ThermoFisher PureLink RNA Mini Kit	TruSeq	NextSeq	2X75	17359223	Qiagen DNeasy	TruSeq	NextSeq	2X150	10016265
Brassicaceae	*Strepthanthus*	*heterophyllus*	ThermoFisher PureLink RNA Mini Kit	TruSeq	NextSeq	2X75	17818482	Qiagen DNeasy	TruSeq	NextSeq	2X150	10220458
Brassicaceae	*Teesdalia*	*nudicaulis*	ThermoFisher PureLink RNA Mini Kit	TruSeq	NextSeq	2X75	59723745	Qiagen DNeasy	TruSeq	NextSeq	2X150	8579441
Brassicaceae	*Thlaspi*	*arvense*	ThermoFisher PureLink RNA Mini Kit	TruSeq	HiSeq	2X100	7651483	Qiagen DNeasy	TruSeq	NextSeq	2X150	6871390
Brassicaceae	*Turritis*	*glabra*	ThermoFisher PureLink RNA Mini Kit	TruSeq	NextSeq	2X75	15406084	Qiagen DNeasy	TruSeq	NextSeq	2X150	10860792
Capparaceae	*Boscia*	sp.	ThermoFisher PureLink RNA Mini Kit	TruSeq	NextSeq	2X75	17980307	Qiagen DNeasy	TruSeq	NextSeq	2X150	10870502
Capparaceae	*Cadaba*	*natalensis*	ThermoFisher PureLink RNA Mini Kit	TruSeq	NextSeq	2X75	18255177	Qiagen DNeasy	TruSeq	NextSeq	2X150	9366417
Capparaceae	Capparaceae	sp.	ThermoFisher PureLink RNA Mini Kit	TruSeq	NextSeq	2X75	17848365	Qiagen DNeasy	TruSeq	NextSeq	2X150	9425035
Capparaceae	*Capparis*	*fascicularis*	ThermoFisher PureLink RNA Mini Kit	TruSeq	NextSeq	2X75	18084217	Qiagen DNeasy	TruSeq	NextSeq	2X150	10378771
Cariaceae	*Carica*	*papaya*	ThermoFisher PureLink RNA Mini Kit	TruSeq	NextSeq	2X75	18870089	Qiagen DNeasy	TruSeq	NextSeq	2X150	13335392

Cleomaceae	*Arivela*	*viscosa*	Qiagen RNEasy	TruSeq	HiSeq	2X100	23245927	Qiagen DNeasy	TruSeq	NextSeq	2X150	9098547
Cleomaceae	Cleomaceae	sp.	Qiagen RNEasy	TruSeq	HiSeq	2X100	25851821	Qiagen DNeasy	TruSeq	NextSeq	2X150	10240576
Cleomaceae	*Cleome*	*africana*	Qiagen RNEasy	TruSeq	HiSeq	2X100	21205456	Qiagen DNeasy	TruSeq	NextSeq	2X150	7267839
Cleomaceae	*Cleome*	*amblyocarpa*	Qiagen RNEasy	TruSeq	HiSeq	2X100	21519053	*NA*	*NA*	*NA*	*NA*	*NA*
Cleomaceae	*Cleome*	*arabica*	ThermoFisher PureLink RNA Mini Kit	TruSeq	NextSeq	2X75	14426573	Qiagen DNeasy	TruSeq	NextSeq	2X150	7960574
Cleomaceae	*Cleome*	*violacea*	ThermoFisher PureLink RNA Mini Kit	TruSeq	HiSeq	2X250	6720824	Qiagen DNeasy	TruSeq	NextSeq	2X150	7683480
Cleomaceae	*Cleomella*	*serrulata*	Qiagen RNEasy	TruSeq				*NA*	*NA*	*NA*	*NA*	*NA*
Cleomaceae	*Coalisina*	*angustifolia*	Qiagen RNEasy	TruSeq	HiSeq	2X100	19351183	Qiagen DNeasy	TruSeq	NextSeq	2X150	8842965
Cleomaceae	*Coalisina*	*paradoxa*	Qiagen RNEasy	TruSeq	HiSeq	2X100	21871851	Qiagen DNeasy	TruSeq	NextSeq	2X150	10046879
Cleomaceae	*Gynandropsis*	*gynandra*	Qiagen RNEasy	TruSeq	HiSeq	2X100	19102136	Qiagen DNeasy	TruSeq	NextSeq	2X150	9236435
Cleomaceae	*Melidiscus*	*giganteus*	Qiagen RNEasy	TruSeq	HiSeq	2X100	19954871	Qiagen DNeasy	TruSeq	NextSeq	2X150	9018300
Cleomaceae	*Polanisia*	*dodecandra*	Qiagen RNEasy	TruSeq	HiSeq	2X100	44674939	Qiagen DNeasy	TruSeq	NextSeq	2X150	8796223
Cleomaceae	*Polanisia*	*graveolens*	Qiagen RNEasy	TruSeq	HiSeq	2X100	25106398	Qiagen DNeasy	TruSeq	NextSeq	2X150	10218176
Cleomaceae	*Polanisia*	sp.	Qiagen RNEasy	TruSeq	HiSeq	2X100	25823291	Qiagen DNeasy	TruSeq	NextSeq	2X150	8242370
Cleomaceae	*Polanisia*	*trachysperma*	Qiagen RNEasy	TruSeq	HiSeq	2X100	38182441	Qiagen DNeasy	TruSeq	NextSeq	2X150	9082194
Cleomaceae	*Sieruela*	*monophylla*	Qiagen RNEasy	TruSeq	HiSeq	2X100	25080383	*NA*	*NA*	*NA*	*NA*	*NA*
Cleomaceae	*Tarenaya*	*hassleriana*	Qiagen RNEasy	TruSeq	HiSeq	2X100	19162929	Qiagen DNeasy	TruSeq	NextSeq	2X150	12209330
Moringaceae	*Moringa*	*oleifera*	ThermoFisher PureLink RNA Mini Kit	TruSeq	NextSeq	2X75	57179455	Qiagen DNeasy	TruSeq	NextSeq	2X150	10239447
Resadaceae	*Ochradenus*	*baccatus*	ThermoFisher PureLink RNA Mini Kit	TruSeq	NextSeq	2X75	18868053	Qiagen DNeasy	TruSeq	NextSeq	2X150	9340353
Resadaceae	*Reseda*	*ordorata*	ThermoFisher PureLink RNA Mini Kit	TruSeq	NextSeq	2X75	18754089	Qiagen DNeasy	TruSeq	NextSeq	2X150	10029969
