# Supplementary material for: Phylogeny and multiple independent whole‐genome duplication events in the Brassicales
Source: Am J Bot. 2020 Aug 24;107(8):1148–64. doi: 10.1002/ajb2.1514 (PMC7496422; doi:10.1002/ajb2.1514)
Supplement: Supplementary file 4 — APPENDIX S4. Orthogroups retained for each analysis. [file AJB2-107-1148-s004.pdf]

Appendix S4. Orthogroups retained for each analysis.

Group	# of genes assigned to groups	% assigned	OrthoGroups	G50	O50	# of groups with all species	Single-copy OrthoGroups	Taxon Occupancy (80%)	Alignment Quality (40% gaps)	# of Trees after first round of RAxML	Tree Pruning (cutoff #)	Final tree # for species tree inference
Brassicales	3516602	96.3	47600	252	4630	6444	0	59/74 = 10968	2663	2663	1284 (10)	1284
Brassicaceae	2224281	96.7	39809	135	5160	7822	0	39/48 = 12129	5587	5587	2110 (10)	2110
Capparaceae	143114	79.4	25179	5	9600	12350	3228	3/4 = 17571	15988	14338	13328 (3)	10214
Cleomaceae	883407	91.9	42218	37	7662	9706	55	13/17 = 14107	10732	10732	3626 (10)	3626
RBMC	165057	78.7	21372	8	7447	9098	881	4/5 = 11756	10038	10038	8476 (4)	8476
