# Supplementary material for: Phylogeny and multiple independent whole‐genome duplication events in the Brassicales
Source: Am J Bot. 2020 Aug 24;107(8):1148–64. doi: 10.1002/ajb2.1514 (PMC7496422; doi:10.1002/ajb2.1514)
Supplement: Supplementary file 5 — APPENDIX S5. BIC scores for 1–4 components for both FASTKs (McKain et al., 2016) and DupPipe (Barker et al., 2010). [file AJB2-107-1148-s005.pdf]

Appendix S5. BIC scores for 1-4 components for both FASTKs (McKain et al., 2016) and DupPipe (Barker et al., 2010) Ks plots.

Species Information			DupPipe BIC Scores				FASTKs BIC Scores			
Family	**Genus**	**Species**	**1 peak**	**2 peaks**	**3 peaks**	**4 peaks**	**1 peak**	**2 peaks**	**3 peaks**	**4 peaks**
Bataceae	*Batis*	*maritima*	-6273.05	-4970.69	-4612.552	-4515.273	-29659.81	-29227.93	-28742.21	-28355.45
Brassicaceae	*Aethionema*	*arabicum*	-17888.73	-15930.41	-15712.96	-15489.97	-72664.46	-71361.21	-70739.96	-70405.8
Brassicaceae	*Barbarea*	*bracteosa*	-20416.34	-18389.58	-18047.26	-17929.21	-55743.7	-54258.87	-53867.64	-53868.36
Brassicaceae	*Cakile*	*maritima*	-49327.51	-48717.18	-48587.63	-48384.6	-140948	-139956.7	-139670.2	-139216.5
Brassicaceae	*Calepina*	*irregularis*	-34320.8	-30254.13	-29871.31	-29871.35	-67744.52	-64841.66	-63941.85	-63706.6
Brassicaceae	*Capsella*	*bursa-pastoris*	-9387.535	-8388.332	-8312.341	-8217.367	-51871.77	-50359.75	-49734.31	-49607.75
Brassicaceae	*Cardamine*	*hirsuta*	-18046.43	-15950.79	-15655.47	-15519.12	-27724.56	-27082.3	-26877.96	-26843.1
Brassicaceae	*Caulanthus*	*amplexicaulis*	-72240.47	-66365.55	-65466.22	-65312.87	-296250.7	-288865.5	-286690	-286481.9
Brassicaceae	*Chorispora*	*tenella*	-14681.18	-13104.49	-12870.93	-12797.43	-27157.47	-26754.78	-26675.19	-26592.95
Brassicaceae	*Cochlearia*	*officinalis*	-26835.57	-25203.31	-24977.02	-24724.24	-45997.75	-45297.29	-44895.66	-44896.44
Brassicaceae	*Crambe*	*hispanica*	-53282.57	-52870.1	-52509.33	-52201.88	-162684.3	-161909.2	-161518.4	-160916.1
Brassicaceae	*Descurainia*	*sophioides*	-20134.61	-17945.48	-17617.56	-17430.33	-78315.54	-76328.78	-74915.19	-74615.81
Brassicaceae	*Descurainia*	*pinnata*	-32177.51	-29224.79	-28697.38	-28298.75	-139705.5	-136187.3	-134855.7	-134853.8
Brassicaceae	*Diptycocarpus*	*strictus*	-20176.44	-18385.49	-18151.68	-18005.14	-51593.24	-49629.33	-49558.3	-49397.94
Brassicaceae	*Eruca*	*vesicaria*	-57302.99	-56279.26	-56171.14	-55940.44	-180363.8	-178936.7	-178670.7	-178002.3
Brassicaceae	*Erysimum*	*cheiranthoides*	-17035.26	-15470.34	-15201.41	-15048.28	-27870.51	-27251.97	-27169.5	-27167.07
Brassicaceae	*Euclidium*	*syriacum*	-7330.347	-6664.564	-6569.126	-6486.873	-75457	-74132.51	-74132.3	-73955.92
Brassicaceae	*Farsetia*	*aegyptia*	-87046.73	-78626.97	-77752.59	-77752.63	-27584.64	-26808.89	-26648.29	-26461.84
Brassicaceae	*Goldbachia*	*laevigata*	-46724.37	-40469.43	-39908.4	-39824.05	-126148.9	-120596.6	-119013.2	-118631.9
Brassicaceae	*Guillenia*	*lasiophylla*	-59735.11	-53526.87	-52969.1	-52857.97	-134706.6	-128952.9	-128027.7	-127946
Brassicaceae	*Hesperis*	*matronalis*	-8981.003	-8402.243	-8296.301	-8279.953	-116152.4	-114019.3	-113171.9	-113171.2
Brassicaceae	*Hirschfeldia*	*incana*	-57478.96	-56124.2	-56066.99	-55528.43	-129935.9	-129659.6	-129543	-128886.2
Brassicaceae	*Iberis*	*amara*	-37998.83	-36103.28	-35797.37	-35513.78	-104300.9	-102190.1	-101688.1	-101659.2
Brassicaceae	*Isatis*	*lusitanica*	-16637.79	-14632.94	-14324.95	-14221.36	-27652.87	-26943.13	-26871.77	-26692.73
Brassicaceae	*Isatis*	*tinctoria*	-32807.62	-28987.6	-28672.49	-28416.86	-64911.3	-62615.86	-61966.98	-61638.4

Brassicaceae	*Lepidium*	*ruderale*	-51478.63	-43198.8	-42721.1	-42559.56	-125387.6	-118218.6	-117136.5	-116627.7
Brassicaceae	*Lepidium*	*sativum*	-63920.94	-57354.73	-56523.84	-56506.45	-235772.5	-226791.3	-224613.5	-224338.9
Brassicaceae	*Lobularia*	*maritima*	-22482.68	-20460.54	-20234	-20233.39	-35962.92	-35163.19	-34845.46	-34845.36
Brassicaceae	*Lunaria*	*annua*	-49086.82	-48726.38	-48391.74	-48324.15	-156452.4	-156020.2	-155609.5	-155183.2
Brassicaceae	*Malcolmia*	*maritima*	-31503.91	-28447.88	-28044.13	-27721.6	-104663.4	-100716.8	-100089.1	-99712.58
Brassicaceae	*Matthiola*	*longipetala*	-18787.75	-16630.62	-16436.26	-16259.59	-28755.13	-28074.03	-27838.22	-27791.81
Brassicaceae	*Meniocus*	*linifolius*	-93350.47	-79319.88	-78028.4	-77924.67	-293255.5	-277515.4	-274443.6	-273907.6
Brassicaceae	*Myagrum*	*perfoliatum*	-18501.89	-16611.93	-16246.46	-16074.25	-46087.16	-44153.4	-43545.81	-43274.85
Brassicaceae	*Nasturtium*	*officinale*	-60471.95	-52138.19	-51393.83	-51165.92	-223823.9	-213019.7	-211035.3	-210681.9
Brassicaceae	*Olimarabidopsis*	*pumila*	-39183.53	-33871.53	-33459.93	-33419.88	-66715.53	-63165.63	-62249.17	-62076.87
Brassicaceae	*Physaria*	*acutifolia*	-41432.66	-40148.94	-40051.83	-39542.31	-100664.9	-100252.2	-100064.3	-99552.98
Brassicaceae	*Psychine*	*stylosa*	-42292.4	-41469.83	-41414.98	-41051.42	-109069.2	-108968.6	-108891.8	-108786.9
Brassicaceae	*Rorippa*	*islandica*	-22644.09	-20601.65	-20221.54	-20067.94	-53588.67	-51903.39	-51623.81	-51592.38
Brassicaceae	*Schizopetalum*	*walkeri*	-42672.93	-41045.12	-40688.12	-40594.02	-134672.9	-133861.7	-132864.5	-132864.6
Brassicaceae	*Sinapis*	*alba*	-58678.87	-58381.11	-57891.62	-57599.67	-214304.1	-213673.6	-213076.5	-212988.6
Brassicaceae	*Sisymbrium*	*brassiciforme*	-56912.82	-51040.69	-50499.11	-50441.32	-106873.4	-106872.7	-106631	-106195.7
Brassicaceae	*Sisymbrium*	*leucocladum*	-18171.84	-16346.14	-16073.08	-15879.77	-29525.35	-28937.77	-28532.62	-28429.9
Brassicaceae	*Sisymbrium*	sp.	-45927.74	-44982.75	-44902.77	-44505.49	-48002.97	-45832.08	-45213.05	-44905.1
Brassicaceae	Brassicaceae	sp.	-92159.93	-85720.01	-84367.26	-84131.4	-397328.4	-390193.2	-386693.6	-385884.6
Brassicaceae	*Strepthanthus*	*arizonicus*	-44070.05	-41193.34	-40805.58	-40760.63	-92667.5	-89997.98	-89422.72	-89310.5
Brassicaceae	*Strepthanthus*	*heterophyllus*	-47146.15	-43765.39	-43355.83	-43327.92	-98277.5	-95744.79	-94996.69	-94933.27
Brassicaceae	*Teesdalia*	*nudicaulis*	-70434.89	-62882.51	-41633.38	-61916.37	-210073	-202074	-199908.6	-199724.9
Brassicaceae	*Thlaspi*	*arvense*	-21362.18	-19322.8	-18939.83	-18772.49	-54264.15	-53094.77	-53083.27	-52492.75
Brassicaceae	*Turritis*	*glabra*	-49465.03	-42282.28	-41683.33	-41633.38	-92297.93	-86449.15	-85442.42	-85345.57
Capparaceae	*Boscia*	sp.	-15462.33	-13656.09	-12905.13	-12735.44	-57128.34	-55238.59	-54170.86	-54008.21
Capparaceae	*Cadaba*	*natalensis*	-10654.85	-8824.122	-8174.672	-8072.744	-47515.81	-45670.41	-45094.29	-44846.13
Capparaceae	*Capparis*	*fascicularis*	-13851.04	-12299.53	-11690.69	-11549.21	-43810.82	-42563.49	-41808.2	-41170.72
Capparaceae	Capparaceae	sp.	-14017.43	-12372.35	-11778.9	-11641.74	-55875.39	-54956.17	-54456.94	-54060.24
Cariaceae	*Carica*	*papaya*	-12964.8	-10810.21	-10361.01	-10318.83	-37739.28	-36667.15	-35950.16	-35652.17

Cleomaceae	Cleomaceae	sp.	-32790.84	-31907.16	-30767.01	-30457.21	-121041.3	-120137.4	-119236.3	-118223
Cleomaceae	*Cleome*	*africana*	-12033.86	-10203.68	-9700.341	-9570.846	-54351.44	-53335.11	-53115.26	-52575.36
Cleomaceae	*Cleome*	*amblyocarpa*	-10307.71	-8534.686	-8097.695	-8023.899	-39832.76	-38657.31	-37793.17	-37665.87
Cleomaceae	*Cleome*	*arabica*	-7773.53	-6276.361	-5974.505	-5878.58	-15333.58	-14779.72	-14518.46	-14386.6
Cleomaceae	*Sieruela*	*monophylla*	-34499.78	-33835.05	-32662.68	-32407.19	-125206.2	-124041.9	-124342.1	-123520.9
Cleomaceae	*Cleome*	*violacea*	-14478.17	-12665.1	-12143.09	-12031.81	-49680.57	-48555.81	-48077.79	-48054.83
Cleomaceae	*Melidiscus*	*giganteus*	-39082.77	-38144.81	-37158.06	-36845.59	-208644	-207264.7	-206914.5	-204958.6
Cleomaceae	*Arivela*	*viscosa*	-12125.13	-10601.82	-10094.69	-9995.74	-46372.67	-44884.62	-44811.79	-44461.78
Cleomaceae	*Polanisia*	sp.	-11321.78	-9636.747	-9186.818	-9072.467	-46405.07	-45258.65	-44304.96	-43969.71
Cleomaceae	*Cleomella*	*serrulata*	-35545.31	-29633.1	-28966.29	-28500.9	-104059.2	-98769.64	-97286.63	-96448.16
Cleomaceae	*Coalisina*	*angustifolia*	-32947.1	-32573.87	-31485.88	-31275.91	-70695.18	-70690.09	-70254.17	-69824.07
Cleomaceae	*Coalisina*	*paradoxa*	-45113.69	-43013.85	-42454.3	-42123.13	-232073.7	-230283.9	-228074.1	-226942.3
Cleomaceae	*Gynandropsis*	*gynandra*	-26445.88	-25136.17	-24368.74	-24136.14	-105952.6	-104635	-103678.3	-103482.8
Cleomaceae	*Polanisia*	*dodecandra*	-15250.73	-13157.53	-12519.87	-12362.36	-62510.34	-61483.52	-60632.61	-60304.93
Cleomaceae	*Polanisia*	*graveolens*	-11921.91	-9855.295	-9345.755	-9212.286	-33712.79	-32733.65	-32274.19	-31957.47
Cleomaceae	*Polanisia*	*trachysperma*	-13878.81	-11855.59	-11252.52	-11116.56	-56577.94	-55015.23	-54726.4	-54212.57
Cleomaceae	*Tarenaya*	*hassleriana*	-34991.25	-34328.86	-33198.46	-33171.58	-113691.1	-113078.4	-112644.8	-112107.3
Moringaceae	*Moringa*	*oleifera*	-8975.359	-6567.789	-6563.584	-6244.213	-19414.45	-18830.46	-18389.46	-18135.18
Resadaceae	*Ochradenus*	*baccatus*	-30000.1	-28976.74	-28050.13	-27748.79	-65666.52	-64702.93	-64643.43	-63693.03
Resadaceae	*Reseda*	*ordorata*	-29667.79	-28450.24	-27615.78	-27382.23	-68356.46	-67711.71	-67638.78	-67120.38
