# Supplementary material for: Phylogeny and multiple independent whole‐genome duplication events in the Brassicales
Source: Am J Bot. 2020 Aug 24;107(8):1148–64. doi: 10.1002/ajb2.1514 (PMC7496422; doi:10.1002/ajb2.1514)
Supplement: Supplementary file 6 — APPENDIX S6. BUSCO analysis of de novo transcriptomes. [file AJB2-107-1148-s006.pdf]

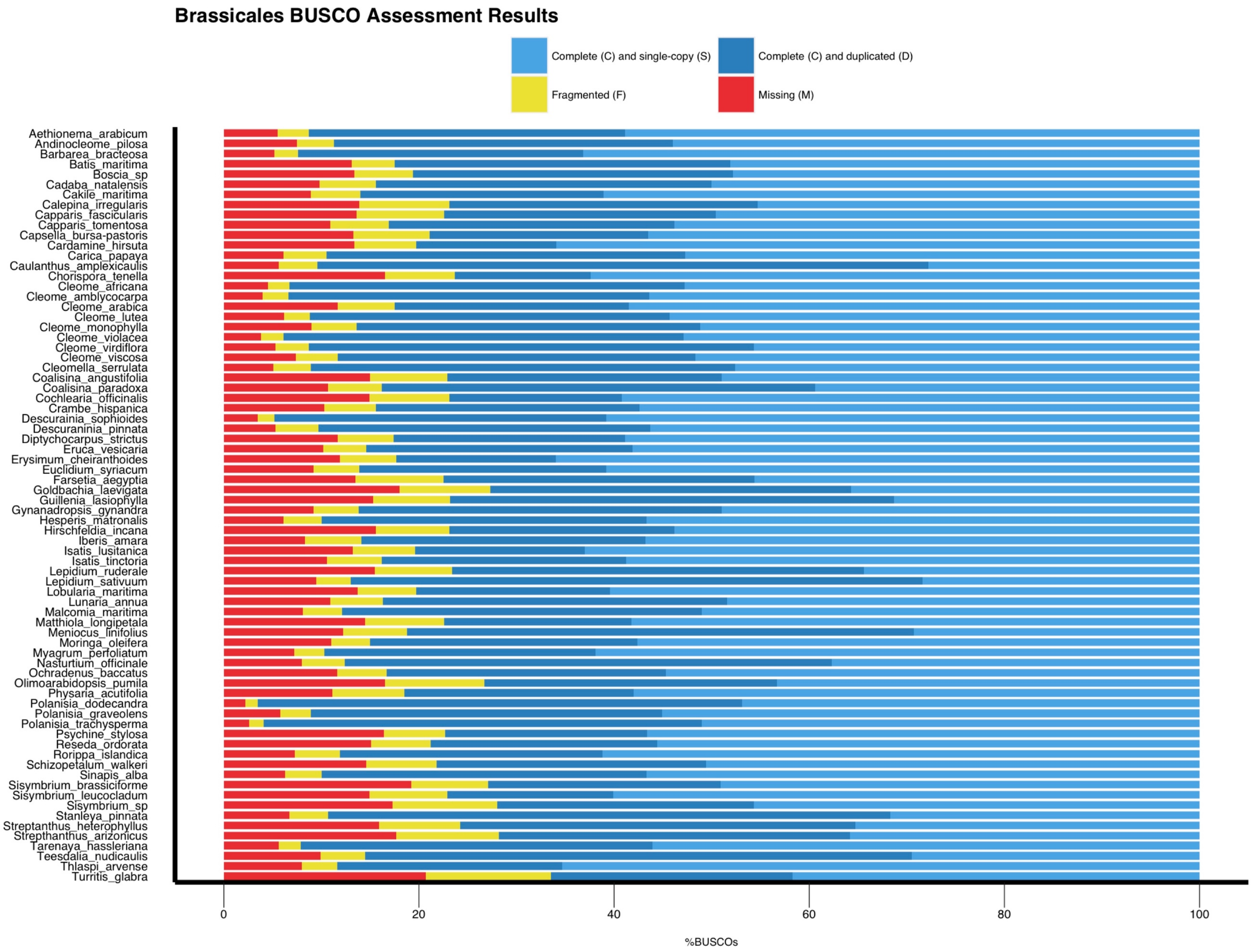

Appendix S6. BUSCO analysis of *de novo* transcriptomes. Legend indicates the percent of genes that are complete and single copy (light blue), complete and duplicate (dark blue), fragmented (yellow), and missing (red) in *de novo* transcriptomes.
