# Supplementary material for: Phylogeny and multiple independent whole‐genome duplication events in the Brassicales
Source: Am J Bot. 2020 Aug 24;107(8):1148–64. doi: 10.1002/ajb2.1514 (PMC7496422; doi:10.1002/ajb2.1514)
Supplement: Supplementary file 7 — APPENDIX S7. Maximum likelihood phylogeny of the Brassicales using two chloroplast genes, MatK and NdhF. [file AJB2-107-1148-s007.pdf]

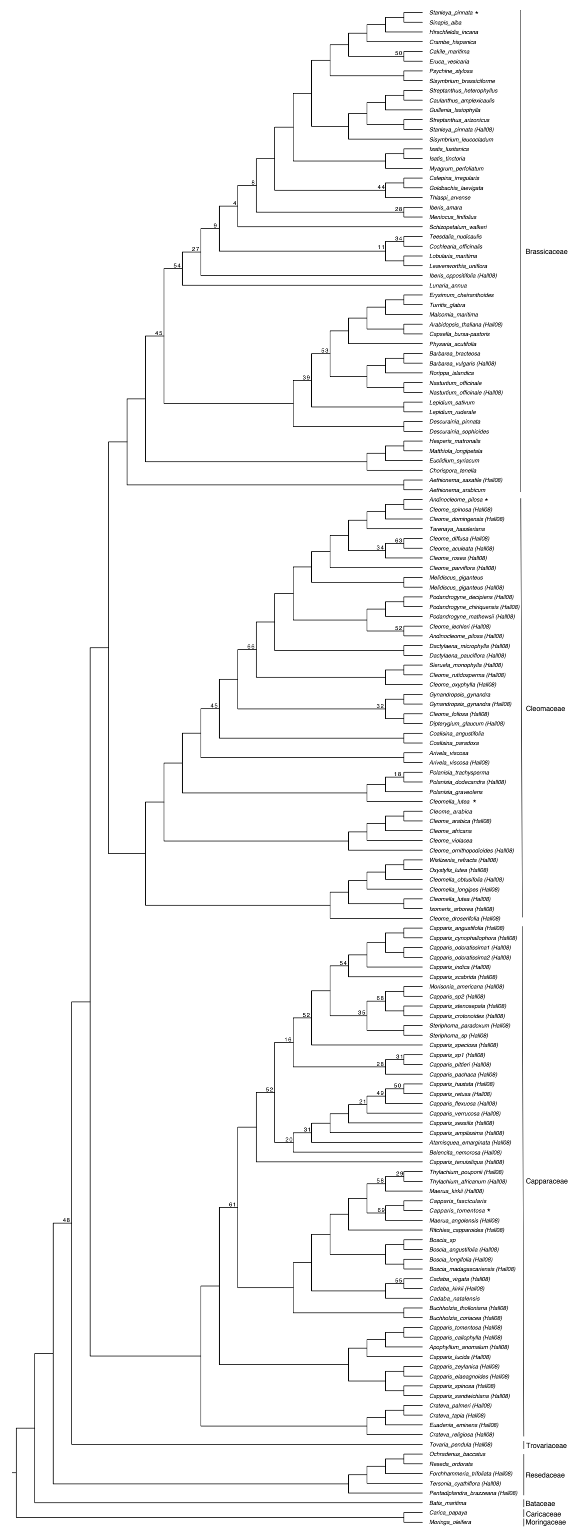

Appendix S7. Maximum likelihood phylogeny of the Brassicales using two chloroplast genes, *MatK* and *ndhF*. Support values are indicated if below 70% bootstrap support. An asterisk (*) indicate taxa whose placement are not sister with samples from Hall (2008).
