# Supplementary material for: Phylogeny and multiple independent whole‐genome duplication events in the Brassicales
Source: Am J Bot. 2020 Aug 24;107(8):1148–64. doi: 10.1002/ajb2.1514 (PMC7496422; doi:10.1002/ajb2.1514)
Supplement: Supplementary file 8 — APPENDIX S8. Maximum likelihood whole chloroplast phylogeny of the Brassicales. [file AJB2-107-1148-s008.pdf]

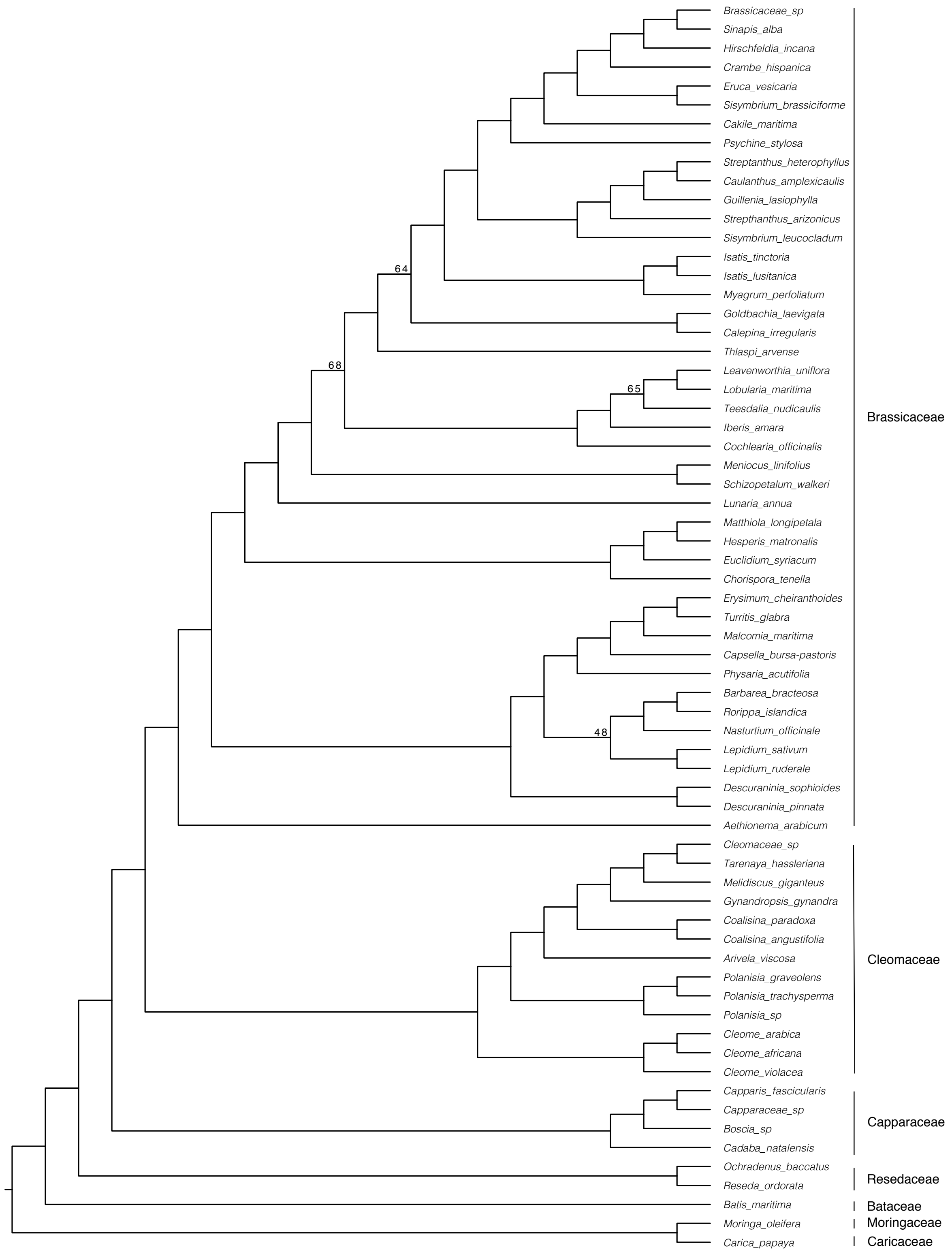

Appendix S8. Maximum likelihood whole-chloroplast phylogeny of the Brassicales. Support values are indicated if below 70% bootstrap support.
