# Supplementary material for: Phylogeny and multiple independent whole‐genome duplication events in the Brassicales
Source: Am J Bot. 2020 Aug 24;107(8):1148–64. doi: 10.1002/ajb2.1514 (PMC7496422; doi:10.1002/ajb2.1514)
Supplement: Supplementary file 9 — APPENDIX S9. Brassicaceae K s plots using both FASTKs (McKain et al., 2016) and DupPipe (Barker et al., 2010). [file AJB2-107-1148-s009.pdf]

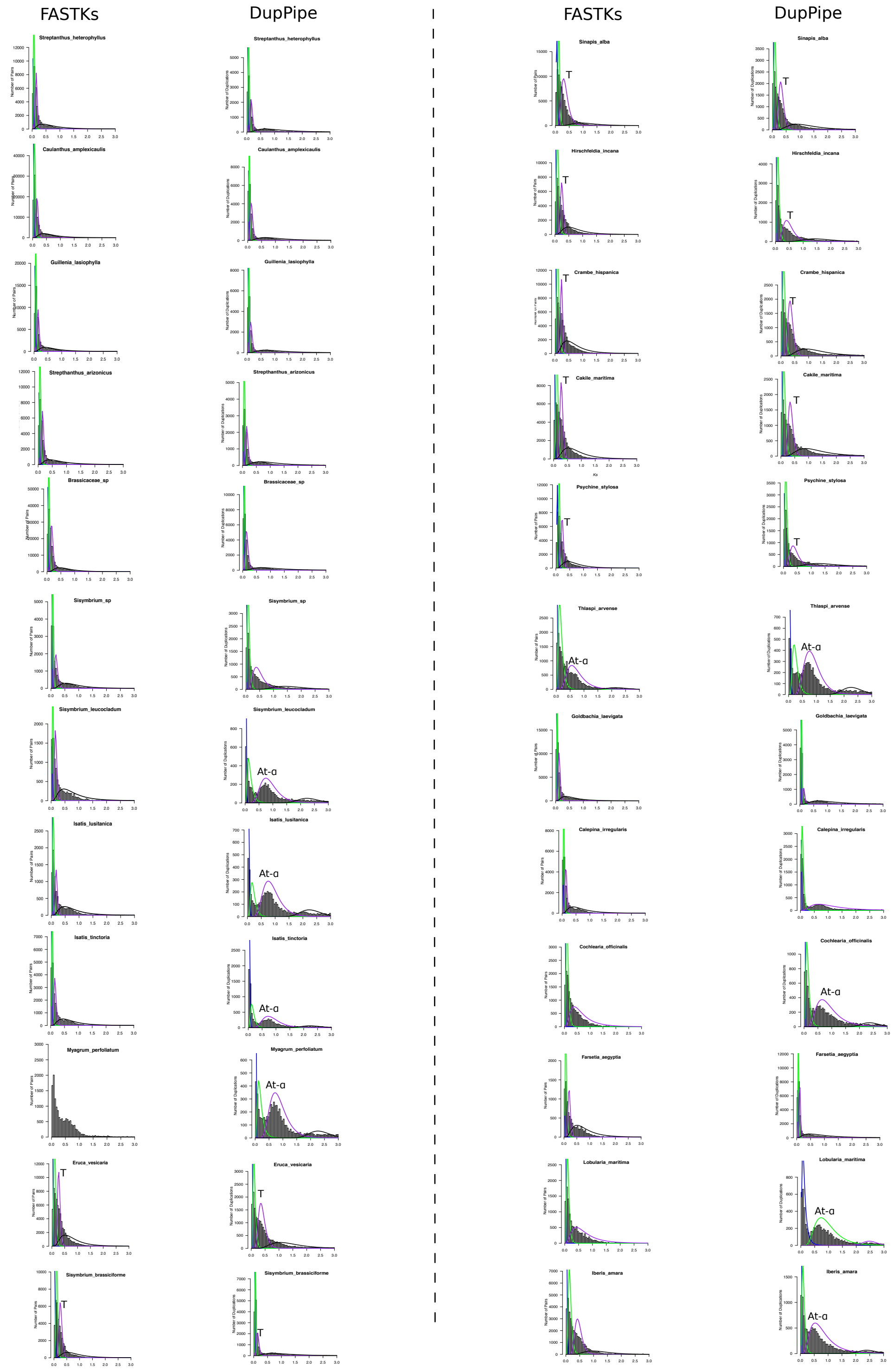
FASTKs
DupPipe
FASTKs
DupPipe
Streptanthus_heterophyllus
Caulanthus_amplexicaulis
Guillenia_lasiophylla
Strepthanthus_arizonicus
Brassicaceae_sp
Sisymbrium_sp
Sisymbrium_leucocladum
Isatis_lusitanica
Isatis_tinctoria
Myagrum_perfoliatum
Eruca_vesicaria
Sisymbrium_brassiciforme
Sinapis_alba
Hirschfeldia_incana
Crambe_hispanica
Cakile_maritima
Psychine_stylosa
Thlaspi_arvense
Goldbachia_laevigata
Calepina_irregularis
Cochlearia_officinalis
Farsetia_aegyptia
Lobularia_maritima
Iberis_amara
T
At-α

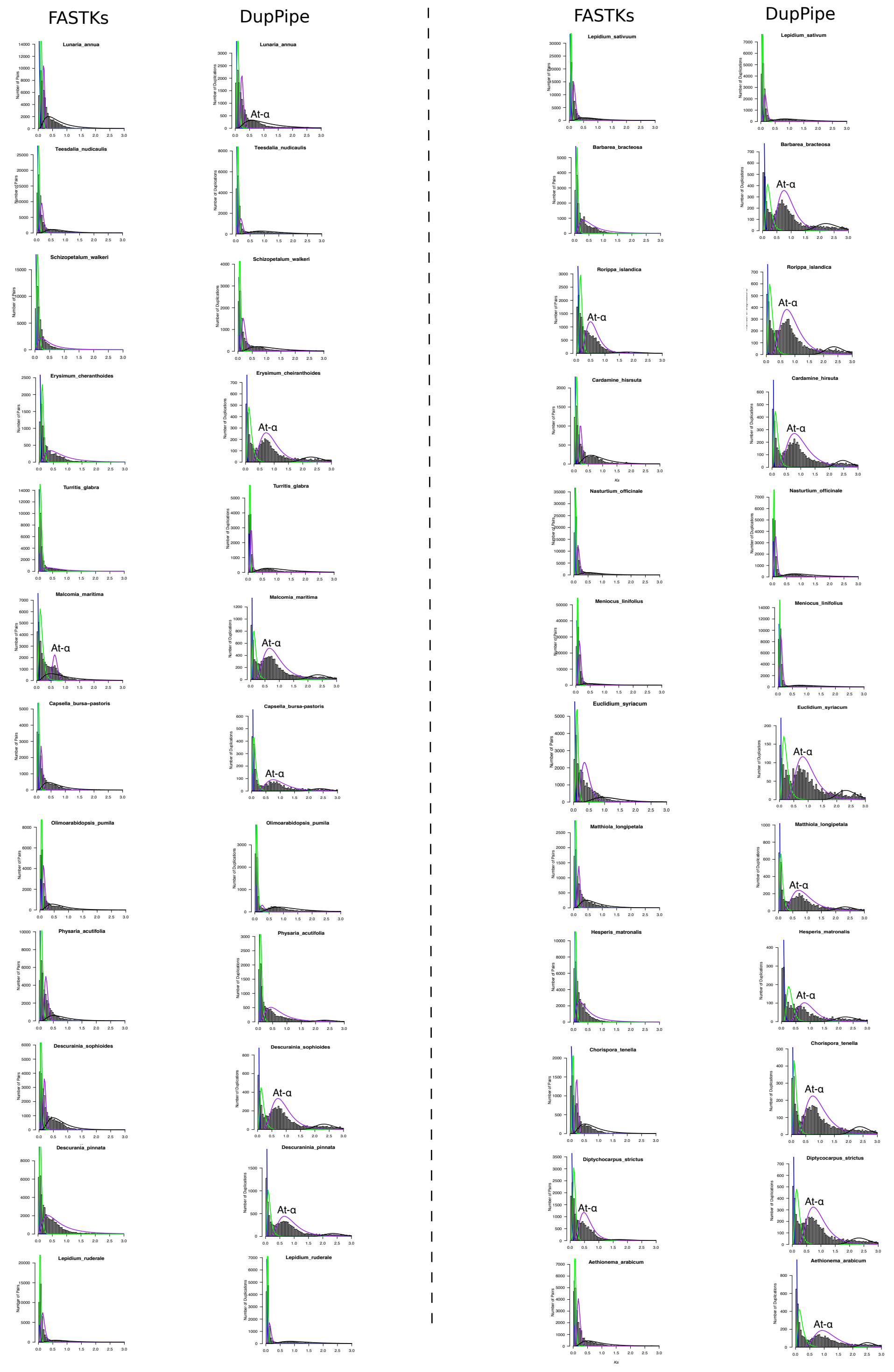

Appendix S9. Ks plots of the Brassicaceae using both FASTKs (McKain et al., 2016) and DupPipe (Barker et al., 2010). Whole-genome duplication events, At-α and the Brassiceae triplication (T) event, are noted above corresponding peaks.
