# Supplementary material for: Phylogeny and multiple independent whole‐genome duplication events in the Brassicales
Source: Am J Bot. 2020 Aug 24;107(8):1148–64. doi: 10.1002/ajb2.1514 (PMC7496422; doi:10.1002/ajb2.1514)
Supplement: Supplementary file 10 — APPENDIX S10. Additional ortholog divergences and K s peaks of the Cleomaceae. [file AJB2-107-1148-s010.pdf]

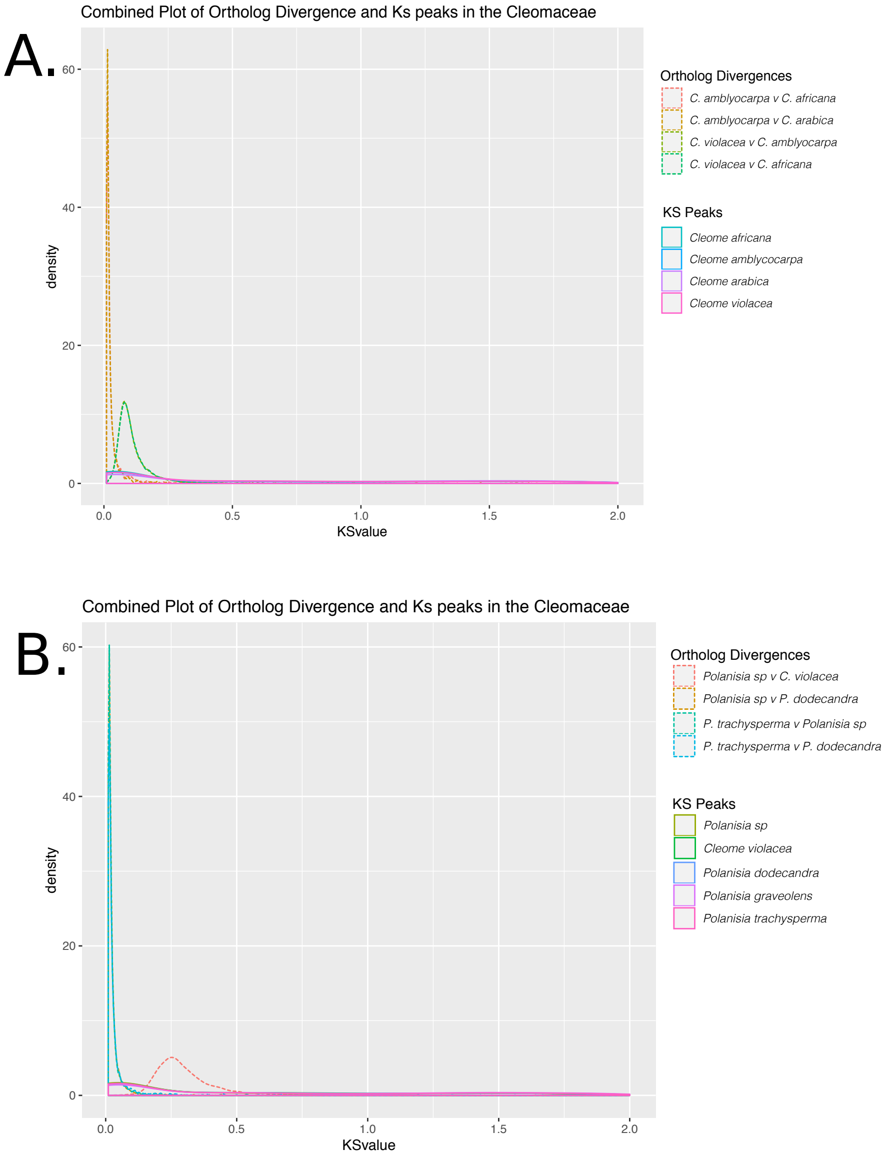

Appendix S10. Ortholog divergences and Ks peaks of the Cleomaceae. **(A)** Ortholog divergences between *C. amblyocarpa* and *C. africana, C. arabica,* and *C. violacea* and between *C. violacea* and *C. africana* to test placement of potential novel WGD event. **(B)** Ortholog divergences between *Polanisia* sp. and *C. violacea, Polanisia* sp., and *P. dodecandra, P. trachysperma,* and *Polanisia* sp. and between *P. trachysperma* and *P. dodecandra* to test for placement of the second potential novel WGD event.
