# Supplementary material for: Phylogeny and multiple independent whole‐genome duplication events in the Brassicales
Source: Am J Bot. 2020 Aug 24;107(8):1148–64. doi: 10.1002/ajb2.1514 (PMC7496422; doi:10.1002/ajb2.1514)
Supplement: Supplementary file 11 — APPENDIX S11. Ortholog divergences and K s peaks of the (A) Capparaceae and (B) Resedaceae + Outgroups. [file AJB2-107-1148-s011.pdf]

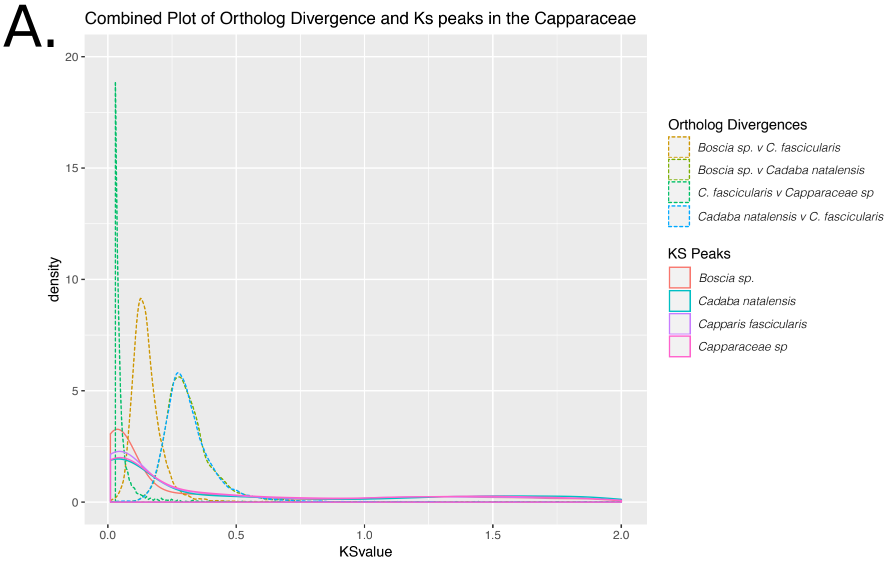

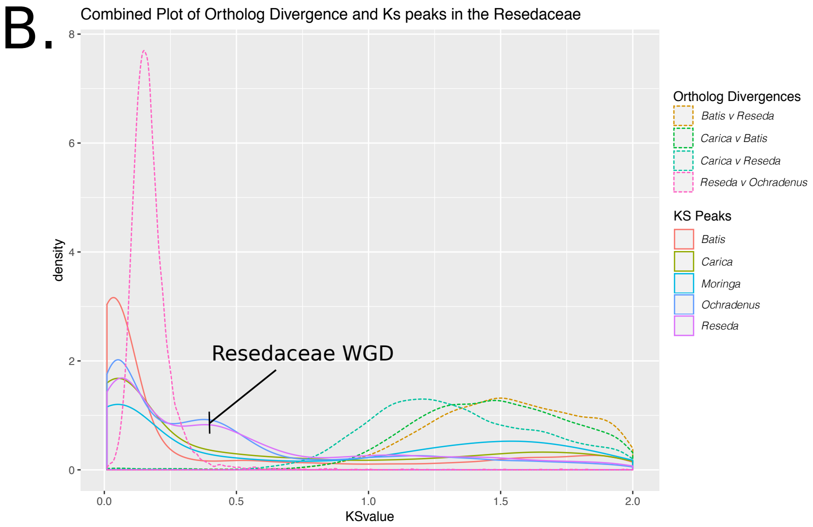

Appendix S11. Ortholog divergences and Ks peaks of the **(A)** Capparaceae and **(B)** Resedaceae + Outgroups. Proposed Resedaceae whole-genome duplication event indicated.
